# Electromagnetic navigation-guided TOES for parapharyngeal tumors: a comparative study on precision and safety

**DOI:** 10.1186/s12893-025-03206-y

**Published:** 2025-10-24

**Authors:** Shihao Wei, Enen Ma, Kexin Zhang, Shoukai Zhang

**Affiliations:** 1 The First Clinical Medicine College, Gansu University of Traditional Chinese Medicine, Lanzhou, 730000 Gansu China; 2 Otolaryngology-Head Neck Surgery, Gansu Provincial Hospital, Lanzhou, 730000 China

**Keywords:** Parapharyngeal space tumors, TOES, ENS, ICA protection, Minimally invasive surgery

## Abstract

**Objective:**

Surgical treatment of parapharyngeal space tumors using traditional methods is associated with a high risk of significant complications, and conventional open surgery has limitations in terms of visualization. This study aims to compare the safety and efficacy of transoral endoscopic surgery combined with the Electromagnetic Navigation system (TOES + ENS) with traditional surgical methods (translateral approach and TOES without ENS) in the treatment of parapharyngeal space tumors.

**Methods:**

We conducted a retrospective comparative analysis of 41 patients diagnosed with parapharyngeal space tumors who underwent surgical resection. Patients were divided into two groups: the TOES + ENS group (T group, *n* = 21) and the traditional surgery group (C group, *n* = 20). Primary outcome measures included surgical duration, intraoperative blood loss, postoperative visual analog scale (VAS) pain scores, time to first oral intake, length of hospital stay, and the incidence and severity of postoperative complications, the latter classified using the Clavien-Dindo system.

**Results:**

Both groups achieved high tumor resection rates, with a complete resection rate of 100% in the T group. In the C group, one case of incomplete partial resection led to recurrence during follow-up. Compared with the C group, the T group demonstrated statistically significant advantages in all perioperative indicators: shorter surgery time (79.10 ± 22.49 min vs. 119.75 ± 17.81 min, *P* < 0.001), reduced intraoperative blood loss (35.43 ± 18.16 mL vs. 92.50 ± 20.93 mL, *P* < 0.001), lower postoperative VAS scores (2.76 ± 0.70 vs. 6.40 ± 1.10, *P* < 0.001), shorter time to oral intake (2.19 ± 0.40 days vs. 3.85 ± 1.42 days, *P* < 0.001), and shorter hospital stay (3.86 ± 1.39 days vs. 7.40 ± 1.10 days, *P* < 0.001). The T group recorded only one transient Clavien-Dindo Grade II complication. In contrast, the C group experienced eight complications, including four severe events: one Grade IIId (permanent nerve damage), one Grade IIIb (vascular injury requiring reoperation), and two Grade IIIa events (abscess requiring drainage).

**Conclusion:**

In this study, although both surgical strategies effectively resected tumors, TOES + ENS was associated with significantly superior safety profiles, characterized by the absence of severe complications and markedly improved postoperative recovery outcomes. Despite the limitations inherent to the retrospective design and small sample size, these findings support TOES + ENS as a valuable and potentially superior surgical option compared to traditional methods for appropriately selected patients with tumors in the parapharyngeal space.

**Supplementary Information:**

The online version contains supplementary material available at 10.1186/s12893-025-03206-y.

## Introduction

The parapharyngeal space is a deep and anatomically complex region in the head and neck, containing critical neurovascular structures, including the internal carotid artery (ICA) and cranial nerves IX through XII. Surgical intervention in this region is highly challenging [1]. In this study, traditional surgical methods include trans-neck approaches and transoral endoscopic tumor resection, which typically require extensive anatomical dissection and are associated with significant risks such as bleeding, nerve injury, and poor functional outcomes. Although TOES has been introduced as a minimally invasive alternative to avoid external incisions, its efficacy is inherently limited when used alone. Endoscopic visualization offers excellent superficial visualization but has poor depth perception, increasing the risk of unintended damage to deep, invisible structures such as the ICA [2, 3].。 These techniques are limited by restricted visualization, high risk of damage to vital structures, and inadequate functional preservation.

In this study, we used ENS to guide tumor resection. The system provides navigation through preoperative three-dimensional reconstruction and intraoperative real-time localization, with a reported localization error of typically less than 2.5 mm. This provides real-time three-dimensional feedback on the distance between the surgical instrument and the internal carotid artery (ICA) (the software system reports an ICA reconstruction error of less than 0.8 mm), thereby establishing a dynamic safety margin and ensuring navigation accuracy within 2.5 mm [4]. The rationale for combining these technologies is to create a synergistic system in which the panoramic view provided by TOES complements the depth navigation safety offered by EN. This integration aims to overcome the limitations of each technology, thereby creating a safer and more precise surgical environment [5, 6]. However, despite these theoretical advantages, there is currently a lack of reliable clinical evidence comparing the combined TOES + ENS method with traditional methods.

Therefore, the primary objective of this study is to conduct a comparative analysis of the clinical efficacy of TOES + ENS and traditional surgical methods in the treatment of parapharyngeal space tumors. We specifically aim to evaluate surgical efficacy, safety characteristics (with a focus on the incidence and severity of complications), and key indicators of postoperative recovery.

## Materials and methods

This study is a retrospective clinical study that has been reviewed and approved by the Ethics Committee of Gansu Provincial People’s Hospital.

This study retrospectively included 41 patients diagnosed with “parapharyngeal space tumors” between September 2017 and October 2024. Among them, 21 patients received TOES + ENS treatment, and 20 patients underwent traditional surgical treatment. The T group included 1 rare case of parapharyngeal space basal cell adenocarcinoma (BCAC).

### Surgeon experience and surgical performance

To ensure consistency in surgical techniques and minimize the impact of surgeon experience on study results, all surgeries in the T group were performed by a single senior surgeon. This surgeon has over 15 years of clinical experience in head and neck tumor surgery, with a specialization in the resection and reconstructive surgery of advanced head and neck tumors, as well as endoscopic skull base surgery. Prior to conducting this study, the surgeon had completed: (1) Over 800 endoscopic nasal cavity-sinus and skull base surgeries independently, including over 150 endoscopic tumor resections involving complex anatomical regions (e.g., pituitary tumors, olfactory neuroblastomas, craniopharyngiomas, etc.). (2) Regularly used ENS or optical neuro navigation systems for over 10 years, with extensive experience in navigation-guided surgery for lesions near critical structures such as the internal carotid artery and optic nerve. (3) Extensive experience in transoral approaches, including numerous tonsil cancer and oropharyngeal cancer radical surgeries.

Surgeries in Group C are also performed by senior head and neck surgeons with equivalent qualifications (over 15 years of experience). This stringent surgeon eligibility criterion is a co.

### Patient grouping and bias control

The decision to opt for TOES + ENS surgery or traditional surgery is made by our multidisciplinary team (MDT) based on a comprehensive assessment of preoperative imaging studies, the relationship between the tumor and critical neurovascular structures, patient preferences, and the surgeon’s experience. To minimize the inherent selection bias inherent in retrospective studies, we conducted a rigorous baseline comparison of key confounding variables that may influence surgical approach selection and outcomes. These variables include: Maximum tumor diameter(cm)༈Abbreviated in the text as “tumor diameter”༉, tumor location (pre/post-stem parapharyngeal space), the minimum distance between the tumor and the ICA as assessed by preoperative imaging (cm), and the integrity of the tumor capsule. The balance of these baseline data was assessed through tests to evaluate the comparability of the two groups of patients before surgery. The parapharyngeal space anatomical location was divided into two regions: anterior and posterior to the styloid process, separated by the myofascial styloid septum. Tumors in the posterior styloid region are considered high-risk due to their proximity to the ICA, internal jugular vein (IJV), and cranial nerves IX–XII [7]. To ensure comparability of baseline characteristics between the two groups, we conducted quantitative assessments of the following key anatomical indicators. First, we measured the shortest linear distance between the tumor margin and the outer membrane of the ICA on standard preoperative Contrast-Enhanced Computed Tomography (CECT). Second, based on the CECT image, we evaluated the imaging characteristics of the tumor and classified it into two categories: “Well-circumscribed” or “Suspicious for Infiltration”. The latter (Suspicious for Infiltration) is based on the fact that the tumor exhibits any or all of the following features on CECT images: 1. Irregular Margins: The tumor borders have lost their smooth, well-defined contours and xhibit lobulation, focal projections, or visible burrs.2.Peritumoral Fat Plane Infiltration: Loss of clear dimensions of normal low-density adipose tissue in the parapharyngeal space immediately adjacent to the tumor or the appearance of striated hyperdensity.3.Satellite Nodules: Separate nodular lesions of similar density to the main tumor mass but located outside its presumed peritumoral plane [8]. It should be emphasized that this imaging-based assessment of “capsular integrity” is intended to quantify and balance the expected surgical separation difficulty at the time of enrollment for the two groups of cases. It is merely a predictive indicator and is a completely different concept from the “marginal status (R0/R1)” assessed in the postoperative pathology report to evaluate the oncological resection effect.

### Learning curve and time bias

To assess the impact of the learning curve and potential time bias on study results, we conducted a subgroup analysis of the two groups of patients based on surgical order. Following the surgical time sequence, we defined the first 5 surgeries in the T group as the “Learning phase” and the remaining 16 as the “Mature phase.” For the C group, which had a longer implementation period and relatively mature techniques, we divided it into the “Early cohort” (first 12 cases, September 2017 to September 2021) and the “Late cohort” (last 8 cases, October 2021 to October 2024).

We compared the surgical duration and intraoperative blood loss within each group across different phases to assess the presence and characteristics of the learning curve. More importantly, we conducted critical intergroup comparisons: we compared the “Learning phase” of Group T with the “Late cohort” of Group T to assess the efficacy of the new surgical technique during its initial application phase; simultaneously, we compared the “Mature phase” of Group T with the “Late cohort” of Group C to evaluate the differences between the two surgical techniques during their respective periods of established proficiency. The design aims to minimize the confounding effects of temporal progression and surgeon experience accumulation.

### Inclusion and exclusion criteria

#### Inclusion criteria

The inclusion criteria for this study focused on the anatomical characteristics of tumors, aiming to assess the feasibility and efficacy of specific surgical approaches. Inclusion decisions were based on preoperative imaging evaluations, and tumors located in the parapharyngeal space, protruding into the oropharynx, and not surrounded by important structures were included. This accurately reflects the clinical decision-making process when the preoperative pathological diagnosis is unclear. Notably, the T group cohort included one case of BCAC confirmed postoperatively as low-grade malignant. The tumor exhibited clear borders and an intact capsule on imaging, with a diameter of approximately 3.0 cm. The surgical treatment principle (requiring complete capsule resection) for this case was indistinguishable from that for the benign tumors commonly encountered in this study (particularly pleomorphic adenomas) in terms of surgical technique. Therefore, the inclusion of this case in the cohort did not introduce heterogeneity in surgical strategy.

#### Exclusion criteria

1. Patients with a history of surgery or radiation therapy on the same side of the head and neck; 2. Patients with preoperative imaging studies confirming extensive tumor invasion of the skull base bone or complete encasement of the ICA (greater than 270 degrees); 3. Patients with known anatomical variations that may significantly increase surgical risk (such as high jugular venous plexus, abnormal internal carotid artery course, etc.); 4. Patients with uncorrectable coagulation dysfunction or who cannot tolerate general anesthesia; 5. Patients who have been diagnosed with highly malignant tumors before surgery require neck lymph node dissection or radical resection.

In addition to routine preoperative examinations, imaging examinations and ENS settings are also required prior to surgery.

### Imaging studies and 3D reconstruction

A prerequisite for performing navigational surgery is the acquisition of high-fidelity 3D anatomical models. In this study, all models were generated based on preoperative imaging data. Our uniform imaging protocol was to perform CECT of the neck in the arterial phase. The arterial phase scan was chosen to maximize the visualization of the ICA and its branches, thus ensuring accurate outlining of its anatomical boundaries. To ensure the high resolution required for reconstruction, a layer thickness of 1.0 mm was used for all CT scans.

**Fig. 1:**
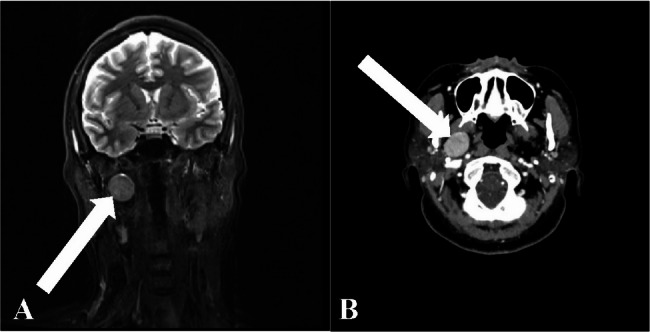
Preoperative Contrast-Enhanced CT and MRI of a Typical T Group Case

To minimize the possibility of subjective errors in outlining anatomical boundaries, we did not leave the segmentation of the tumor and adjacent key structures to a single operator. We established a dual operator quality control protocol for this purpose. The process was as follows: a senior resident and an attending physician independently created 3D models from the CECT data. Subsequently, the two models were aligned and compared. If either anatomic boundary differs by more than 1.0 mm, the two physicians will jointly review the original images to agree on a final, accurate anatomic contour. This cross-validation method is designed to reduce inter-observer variability, thereby enhancing the structural fidelity of the final model used for surgical navigation. Figure 1 demonstrates typical MRI and CECT images of a parapharyngeal interstitial tumor suitable for this procedure.

#### ENS setup

Preoperative imaging data is obtained, and a three-dimensional anatomical model is reconstructed with precise marking of important structures (such as the ICA) to provide clear guidance for intraoperative navigation.

A: MRI (coronal view) demonstrates an abnormal signal lesion (1.8 × 2.3 cm) posterior to the right medial pterygoid muscle. B: Contrast-enhanced CT (axial view) shows an abnormal signal lesion (2.0 × 2.4 cm) posterior to the right medial pterygoid muscle.

### Anesthesia management

All patients undergoing TOES + ENS surgery were managed using a standardized anesthesia protocol. All patients underwent general anesthesia via endotracheal intubation. Patients were positioned in the supine position, with the head rigidly fixed using a Mayfield three-point head frame to ensure absolute stability of the head position throughout the navigation surgery, which is a prerequisite for maintaining navigation accuracy.

### Surgical equipment

Group T: The Stryker 590 H endoscopic system equipped with a rigid endoscope was used in combination with the Medtronic Stealth Station S8 navigation system (StealthStation: 8.0) for the procedure. All surgical procedures were recorded in high-definition video format for subsequent analysis. Conventional Surgery Group: Conventional neck surgery instruments were used, along with the same endoscopic system as in the T Group. For complex cases, the Leica M530 OHX surgical microscope was employed to enhance visual clarity. The surgical procedures were also recorded via video for archiving. This study was conducted using the StealthStation S8 navigation system throughout. To ensure consistency and comparability of study results, we established strict standardized operating procedures for the equipment. During the study period (from October 2021 to October 2024), no major hardware replacements that could affect navigation accuracy were performed on the system.

Prior to each surgery, we performed the standard system calibration procedure. Also, a rigorous accuracy verification process is performed prior to surgery to ensure the accuracy of the ENS and the safety of the patient’s procedure. This standardized process ensured that all enrolled cases were completed under the same precise navigation performance, minimizing potential bias in study results that could arise from fluctuations in device status.

### Navigation system quality assurance protocol

To ensure the highest precision and safety during surgical navigation, this study followed a standardized quality assurance protocol. (1) Preoperative verification: After importing the patient’s Digital Imaging and Communications in Medicine (DICOM) data into the navigation system, two physicians independently verified the consistency of the three-dimensional reconstruction model (especially the internal carotid artery, ICA) with the original images. (2) After the patient is under general anesthesia, the electromagnetic navigation system’s field generator is fixed at the corresponding position on the patient’s head. We use a multi-point matching surface registration method, employing a probe with sensors to sequentially touch and align multiple stable, distinct bony anatomical landmarks on the patient’s face. The registration process is repeated until the system reports a Target Registration Error(TRE)stable below 2.5 mm, at which point the registration is accepted, and the surgery can begin. (3) Real-time accuracy verification: After completing the initial patient-image alignment, we do not immediately begin tumor resection. Instead, we must perform a mandatory, two-phase validation process to confirm the accuracy of the navigation system in real time. The first stage is a global accuracy check. This step is designed to confirm the macroscopic accuracy of the overall registration. Using the tip of the navigation probe, the operator will touch a stable, easily accessible bony landmark point away from the surgical area, specifically the anterior surface of the patient’s frontal bone. This initial validation step is detailed in Supplementary Fig. 1.The second stage is a more critical local accuracy check, which focuses exclusively on the surgical area. We recognize that there may be spatial variations in the accuracy of navigation, so this step is critical. Prior to initiating tumor isolation, we gently touch the rigid anatomic landmarks immediately adjacent to the parapharyngeal space, such as the medial pterygoid plate or the hard palate, with the tip of the probe. The purpose of this action is to ensure that the virtual probe position on the screen corresponds precisely to the real position of the probe touching these localized structures. Only after the accuracy of this local examination has been confirmed to be reliable and stable, the tumor resection will officially begin. This two-stage validation protocol provides us with double confidence: both in the accuracy of the overall system setup and (and this is crucial) in the localized accuracy of the model in the region immediately adjacent to the critical neurovascular structures. (4) Troubleshooting: If significant errors persist, the contingency plan is activated, switching to traditional endoscope-guided or open surgery as an alternative. Safety is always the top priority.

### Surgical procedures

**Fig. 2:**
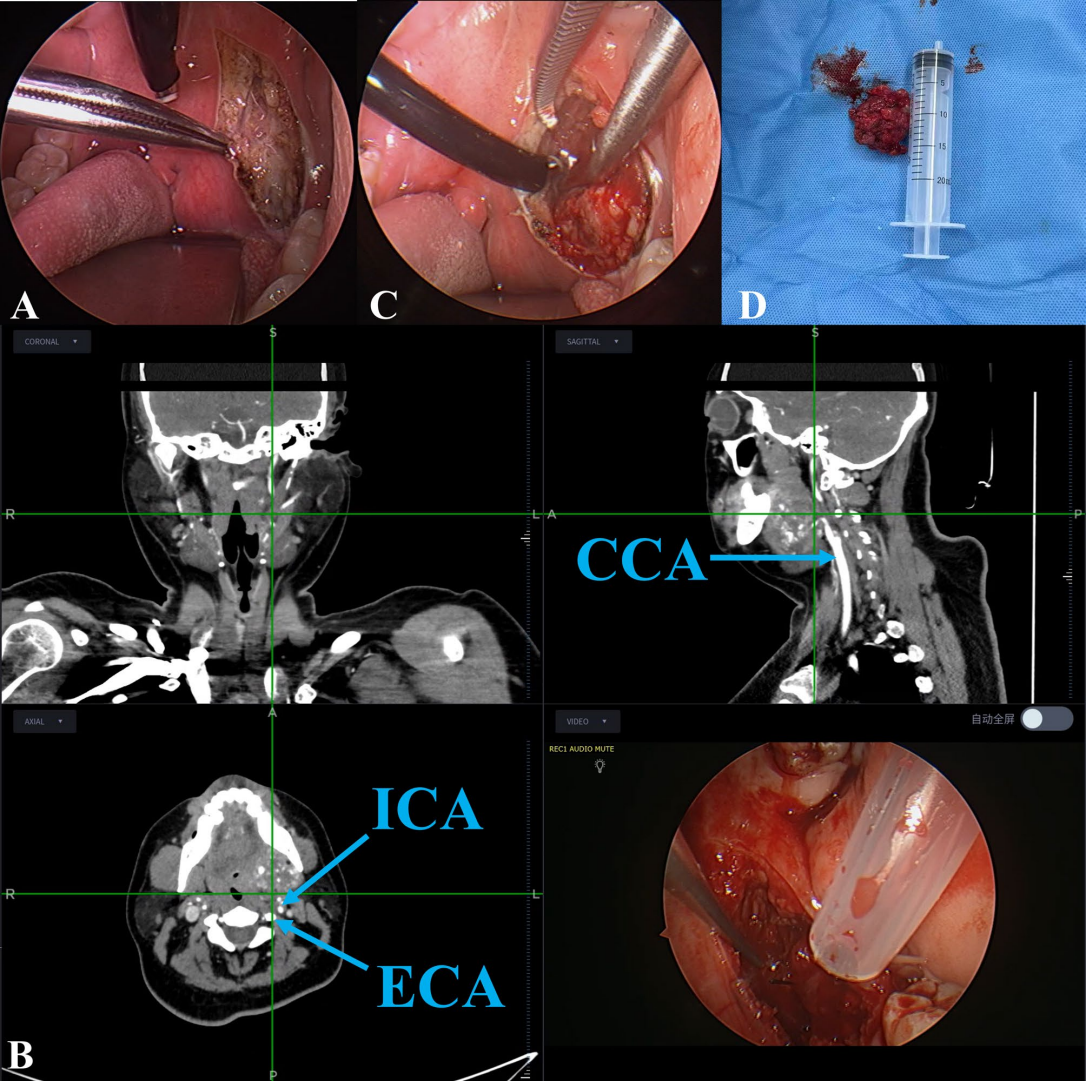
Surgical procedure flowchart

Group T: All patients underwent general anesthesia and endotracheal intubation via the mouth, and were placed in the supine position with the head extended backward and the shoulders supported. An oral retractor was used to open the mouth and expose the surgical field. A 0° or 30° endoscope was inserted to observe the oral cavity, while ENS was activated. For registration, four specific and stable anatomical landmarks were identified on the preoperative CT images and subsequently marked on the patient. These points were: (1) the midpoint of the hard palate; (2, 3) the most inferior points of the bilateral pterygoid process bases; and (4) the gonion (the angle of the mandible) on the side of the lesion. The selection of these non-collinear points was designed to ensure high-fidelity registration and minimize rotational error. The mucosa and submucosa were incised medially to the pterygomandibular ligament down to the pharyngeal constrictor muscle layer to access the parapharyngeal space. Under real-time navigation monitoring, the distance between the tumor and critical structures (e.g., ICA, vagus nerve) was dynamically tracked. A plasma knife was used to dissect precisely along the tumor capsule to ensure safety. Soft tissues were dissected until the loose tumor capsule was identified, and the tumor was gradually enucleated from the easier-to-separate end. If tough tissues were encountered during capsule dissection, sharp mechanical forceps were used cautiously to avoid capsule rupture and tumor spillage; tumor debulking was performed when necessary to facilitate safe and efficient dissection. After complete tumor resection, suture and drain placement were decided based on the surgical cavity condition (Fig. 2).

A Transoral approach: Using an electrosurgical knife, incise the posterior pharyngeal wall mucosa along the edge of the soft palate to expose the tumor.
B Intraoperative global view showing synchronized integration of the endoscopic image and real-time navigation interface, Intraoperative real-time navigation interface displaying the relative positions of the tumor, ICA, ECA, and CCA. The crosshair indicates the tip of the instrument to ensure safety.
C Endoscopic view, with the tumor capsule being carefully dissected under navigation guidance.
D The resected tumor specimen, with its size compared to a 20ml syringe.

### Observation indicators

Surgical-related: Surgical duration (min), intraoperative blood loss (ml); Safety: Nerve injury rate, vascular injury (defined as intraoperative blood loss ≥ 100 ml), surgical cavity effusion or infection (defined as postoperative CT showing fluid shadow area > 5 ml or positive bacterial culture), and functional preservation rate (assessed by permanent or temporary hoarseness, dysphagia, etc.); Postoperative recovery-related: Postoperative hospital stay (days), time to first oral intake (days), postoperative 24-hour VAS score (International Association for the Study of Pain (IASP)-recommended standardized 10-cm visual analog scale; 0 indicates no pain, and 10 indicates the most severe imaginable pain), and wound healing outcomes [classified into three grades (A, B, C) according to standardized surgical criteria, independently assessed by a panel of ≥ 2 senior attending physicians on the 7th postoperative day]. Neither group underwent tracheostomy.

## Statistical methods

Statistical analysis and graphical design were performed using SPSS 27.0 and RStudio 3.0.1 software. Continuous variables that followed a normal distribution were expressed as mean ± standard deviation (Mean ± SD) and analyzed using an independent samples t-test; continuous variables that did not follow a normal distribution were expressed as median and interquartile range (Median [IQR]) and analyzed using the Mann-Whitney U test. Categorical variables were analyzed using chi-square tests or Fisher’s exact tests. To further quantify the balance of baseline variables between the two groups, we calculated the standardized mean difference (SMD). Generally, an SMD ≤ 0.2 indicates that the difference between the two groups is clinically negligible. To account for multiple comparisons among the primary outcome variables, a Bonferroni correction was applied, and a p-value of < 0.01 was considered statistically significant. To assess postoperative tumor recurrence, we employed the Kaplan-Meier method to plot and analyze the recurrence-free survival rates of the two patient groups and used the Log-rank test to perform statistical comparisons of the survival curves. All statistical analyses were performed using SPSS 27.0 software, and *P* < 0.05 was considered statistically significant. To assess the independent effect of surgical approach, we established multivariate linear regression models for five key clinical outcome measures (surgical time, length of hospital stay, postoperative 24-hour VAS score, time to resume oral diet, and intraoperative blood loss) to account for potential confounding factors. In all models, we included group assignment (T group vs. C group) as the primary independent variable and adjusted for patient age (years), tumor diameter (cm), and tumor location (pre-stem space/post-stem space) as covariates. The significance level α was set at 0.05.

Since tumor diameter is an important influencing factor, this study used statistical analysis to compare the effects of grouping (T group vs. C group) on the outcome variables (surgical time/blood loss), while controlling for continuous covariates (tumor diameter). First, we tested the interaction effect between Group and tumor diameter to validate the core assumption of ANCOVA—homogeneity of regression slopes. After confirming that this assumption was met (interaction term *p* > 0.05), we performed a one-way analysis of covariance. We assessed the main effect of Group on surgical time (or blood loss), calculating the adjusted mean values and standard errors (SE) for each group. In addition, we conducted a post-hoc power analysis to assess the statistical power of this study to detect differences in the rate of neurological damage with the current sample size. At the same time, we estimated the prospective sample size required to achieve 80% test power in future studies. All power analyses were performed using G*Power 3.1 software, with a significance level (α) set at 0.05.

Although we believe that this case of low-grade malignant tumor is comparable to benign tumors in terms of surgical treatment, we still conducted a sensitivity analysis to completely rule out the possibility of it being a potential confounding factor from a statistical point of view. In this analysis, we excluded this patient from the T group and re-conducted comparisons of all primary outcome measures between the two groups using the remaining case data.

## Results

### Demographic characteristics

**Fig. 3:**
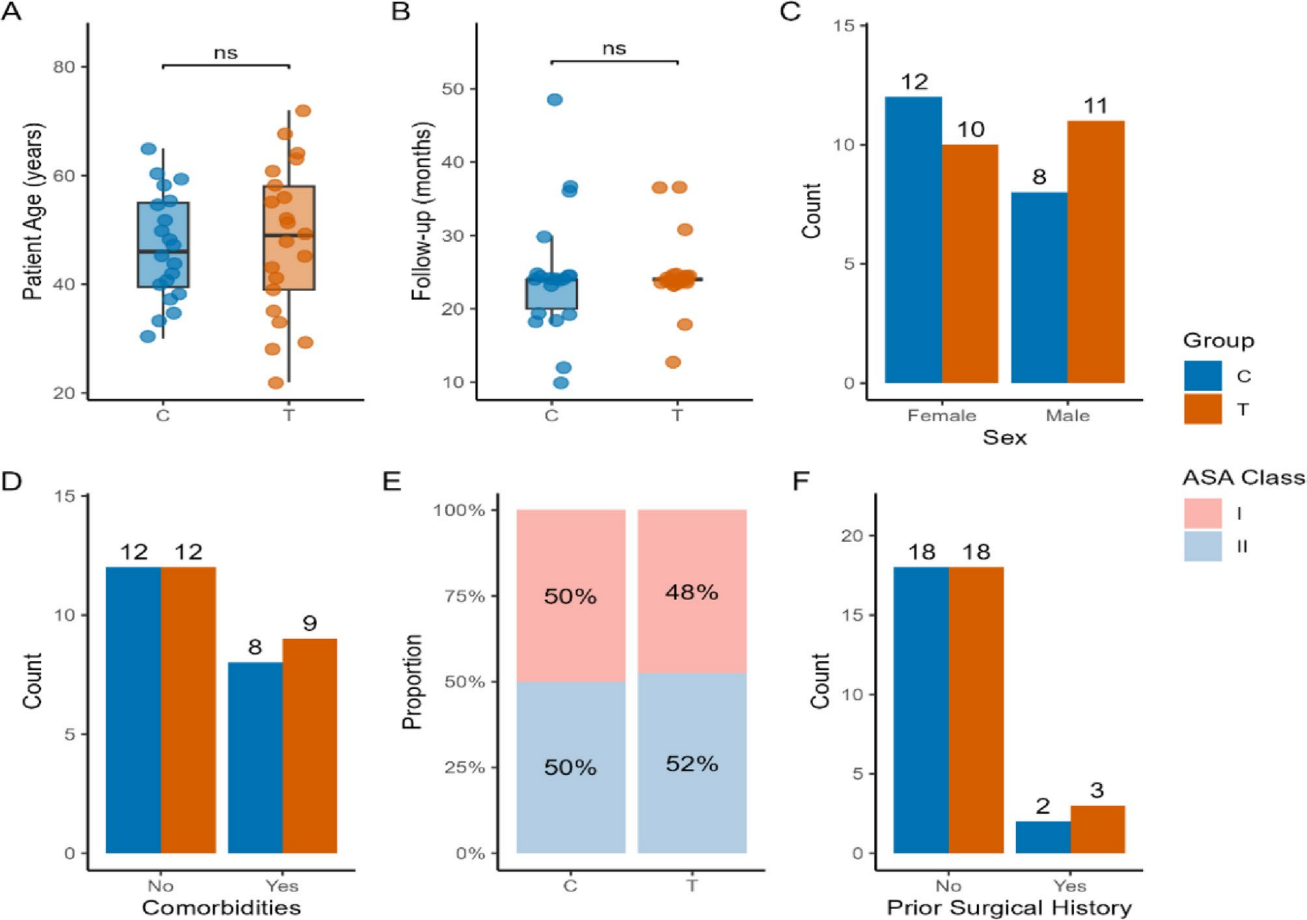
Baseline indicator 1

In terms of gender, compared with Group C, 52% of participants in Group T were male (52% vs. 48%), with no significant difference between the two groups (*P* = 0.536). In terms of age, the mean age in Group T was 48.19 ± 13.89 years, and the mean age in Group C was 46.70 ± 9.87 years, with no significant difference between the two groups (*P* = 0.696). The average follow-up duration was 24.57 ± 4.99 months (1–48 months) in Group C and 24.40 ± 8.40 months (1–36 months) in Group T, with no significant statistical difference between the two groups (*P* = 0.496). (As shown in Fig. 3)

### Histopathologic features of tumors

**Fig. 4:**
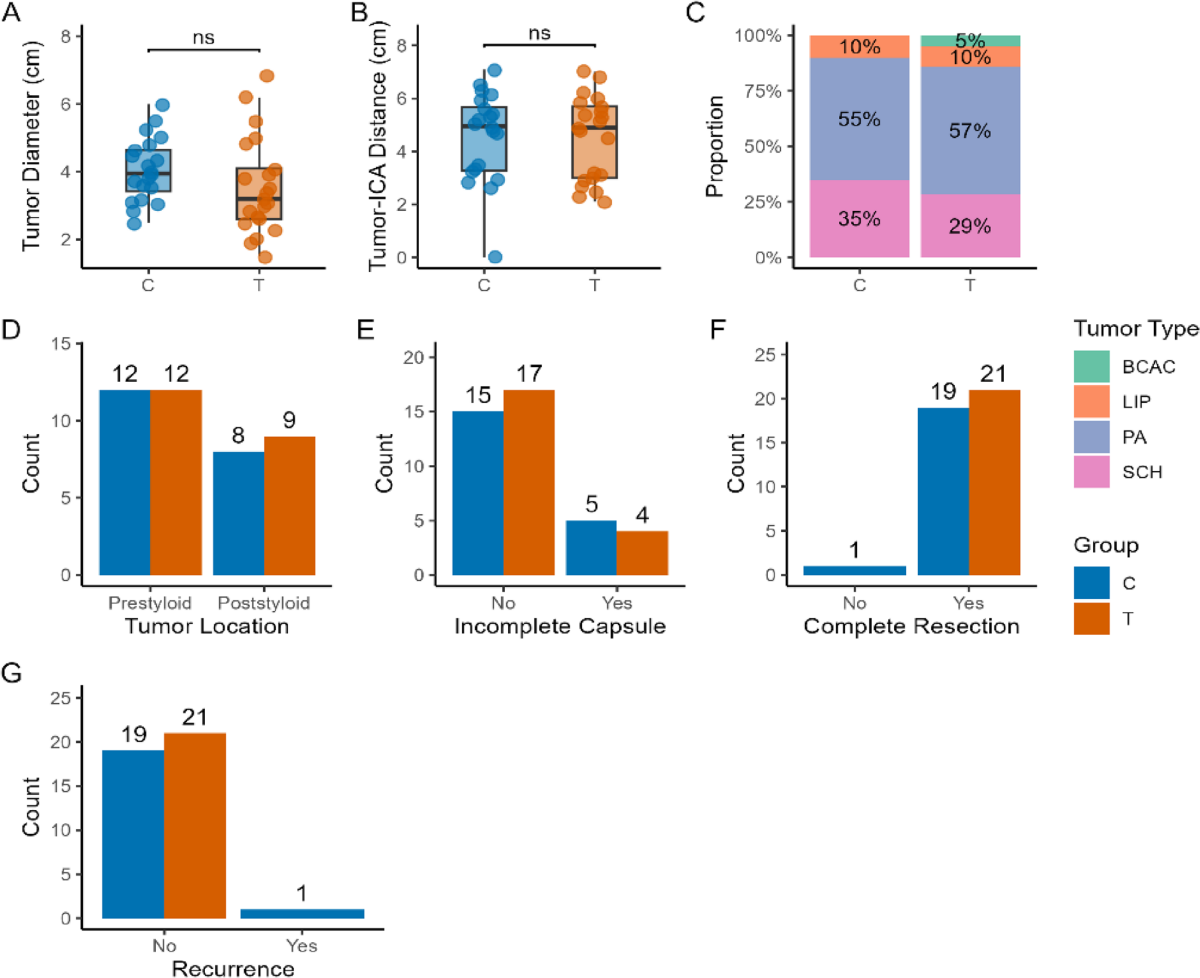
Baseline indicator 2

The most common pathological type was pleomorphic adenoma (PA) [12 cases (57.1%) in the T group vs. 11 cases (55.0%) in the C group]; followed by schwannoma (SCH) [6 cases (28.6%) in the T group vs. 7 cases (35.0%) in the C group]; lipoma (LIP) [2 cases (9.5%) in the T group vs. 2 cases (10.0%) in the C group]. The T group included 1 case of BCAC, while there were no malignant cases in the C group. (As shown in Fig. 4)

### Important prognostic influences

Regarding key prognostic factors, the two groups showed good balance (except for tumor diameter). Specifically, the T group and C group had similar minimum distances between the tumor and the internal carotid artery (ICA) (4.51 ± 1.53 mm vs. 4.56 ± 1.67 mm, SMD = 0.03, *P* = 0.763); the proportion of incomplete capsules (19.0% vs. 25.0%, SMD = 0.02, *P* = 0.619); tumor diameter (3.55 ± 1.42 cm vs. 4.06 ± 0.94 cm, SMD = 0.42, *P* = 0.187); Complications: There was no significant statistical difference between the T group (12 cases without complications, 1 case of hyperlipidemia, 3 cases of type 2 diabetes mellitus, 4 cases of hypertension, and 1 case of hypothyroidism) and the C group (12 cases without complications, 1 case of hyperlipidemia, 3 cases of type 2 diabetes mellitus, and 4 cases of hypertension) (*P* = 0.96); Regarding ASA classification, there was no significant statistical difference between the T group (II: 11 cases, I: 10 cases) and the C group (II: 10 cases, I: 10 cases) (*P* = 1.000); Regarding surgical history, there was no significant statistical difference between the T group (1 case each of appendectomy, septoplasty, and cholecystectomy, with the remainder having no surgical history) and the C group (1 case each of knee arthroscopy and tonsillectomy, with the remainder having no surgical history) (*P* = 1.000). (As shown in Fig. 4)

### Complete resection rate and recurrence rate

Compared with Group C, the complete resection rate in Group T was [100% (21/21) vs. 95.0% (19/20)], with no significant statistical difference between the two groups (*P* = 0.488). The recurrence rate in Group T was 0% (0 cases), while that in Group C was 5.0% (1 case, a pleomorphic adenoma that recurred 3 years postoperatively). There was no significant statistical difference between the two groups (*P* = 0.488). (As shown in Fig. 4) 

###  Oncology efficacy

This study included a total of 41 patients with parapharyngeal space tumors, including 21 cases in the T group and 20 cases in the C group. We confirmed the oncological adequacy of all malignant and borderline tumors. In terms of the thoroughness of tumor resection, the T group performed exceptionally well, with all cases achieving microscopically negative margins (R0) and a complete resection rate of 100%. Among these, the closest margin distance for this case of BCAC was 1 mm. When evaluating more refined capsular integrity metrics for benign tumors, we found that for all tumors with intact capsules as shown on preoperative imaging, the technique was able to preserve the capsule intact and resect the tumor en bloc during surgery. Notably, even for a small number of challenging cases where preoperative imaging had already indicated capsule discontinuity, the precise guidance of ENS still enabled surgeons to dissect at the level of healthy tissue outside the tumor, thereby achieving oncologically radical resection, without causing any new iatrogenic capsule rupture or tumor dissemination during surgery.

These results collectively indicate that the TOES + ENS technique not only ensures oncologically safe margins but also provides excellent anatomical protection of the tumor capsule, which is of significant importance for reducing the risk of postoperative recurrence in benign tumors.

### Analysis of complications

**Fig. 5:**
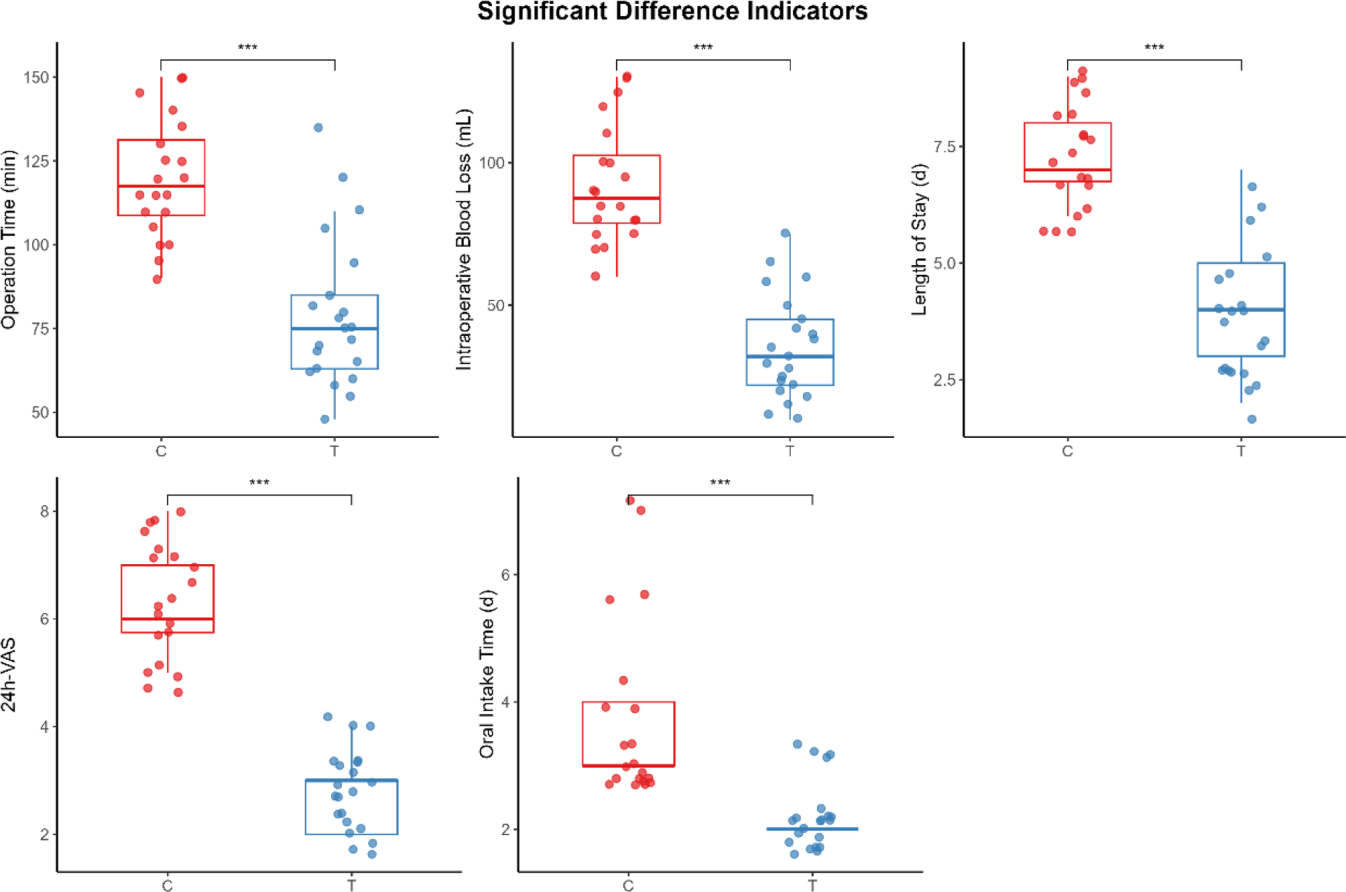
Significant Difference indicators

Group C complications: A patient with a pleomorphic adenoma experienced severe adhesion of the cyst wall, leading to cyst wall rupture and resulting in permanent damage to the hypoglossal nerve. Three years later, residual cyst wall material caused local recurrence. A patient with a schwannoma sustained damage to a branch of the external carotid artery during surgical dissection, necessitating immediate vascular repair. Postoperatively, poor drainage led to seroma formation in the surgical cavity, ultimately resulting in Grade C wound healing (revised after secondary debridement on the seventh postoperative day). T Group Complications: We observed one postoperative complication. The patient had a schwannoma, and intraoperative exploration revealed tight adhesion between the tumor and the pharyngeal branch of the vagus nerve. Despite meticulous dissection and identification of the nerve trunk under real-time ENS guidance and nerve monitoring, and successful complete tumor resection, immediate bedside fiberoptic laryngoscopy revealed restricted mobility of the left vocal cord, indicating incomplete paralysis. We inferred that the injury mechanism was temporary neuropraxia caused by the inevitable traction of the nerve branches during tumor separation, rather than thermal injury or transection. Immediate treatment and management: We immediately administered nebulizer inhalation therapy to the patient and instructed them to refrain from speaking and reduce vocalization. At the same time, to reduce local edema, we administered short-term steroid therapy with dexamethasone. The patient did not experience significant aspiration or dyspnea. Follow-up and recovery: We performed two fiberoptic laryngoscopy examinations on the patient on the third and seventh days after surgery. On the third day after surgery, there was partial improvement in vocal cord mobility. By the seventh day after surgery, the affected vocal cord had fully recovered, with clear and strong voice production, indistinguishable from preoperative status. This rapid and complete recovery process also confirmed our assessment that the injury mechanism was temporary nerve dysfunction. A patient with a pleomorphic adenoma, with a tumor diameter of 6.8 cm, underwent nasal negative pressure drainage to manage minor surgical wound drainage, achieving Grade A wound healing (not classified as a complication). (As shown in Fig. 5).

A rare case of BCAC (Ki-67 10%, low MIB-1 index) was treated with a 1 cm margin-extended resection guided by the Medtronic StealthStation^®^ ENS. No neurovascular injury or involvement of critical structures occurred. MRI at 24 months postoperatively showed no recurrence, and swallowing and voice functions were well preserved postoperatively. No other special complications were observed in either group.

### Indicators of significant advantages of TOES + ENS technology

**Fig. 6:**
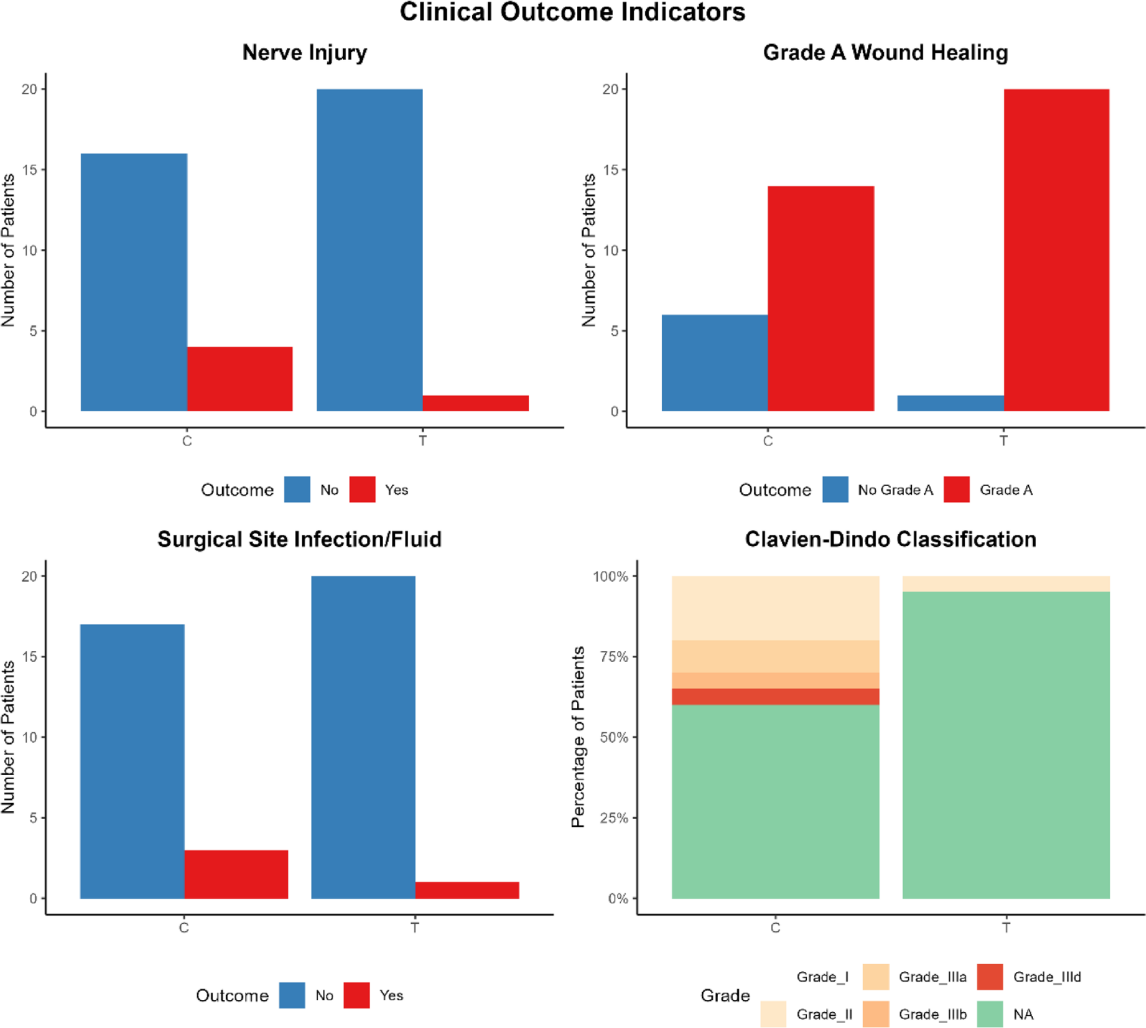
Clinical Outcome Indicators

To enhance the reliability of the results, we applied Bonferroni correction (*n* = 5, α’=0.01) for the following indicators to control the risk of Type I error. The T group demonstrated superior outcomes in the following indicators: intraoperative blood loss [(35.43 ± 18.16) mL vs. (92.50 ± 20.93) mL, *P* < 0.001], with an average difference of −50 mL between the two groups, and a 95% confidence interval for the difference ranging from − 55 mL to −45 mL (*P* < 0.001); surgical time (including endoscopic system localization, navigation data collection, and tumor resection) [(79.10 ± 22.49) min vs. (119.75 ± 17.81) min, *P* < 0.001], with an average difference of −34 min between the two groups, and a 95% confidence interval for the difference ranging from − 41 to −27 min (*P* < 0.001); Length of hospital stay [(3.86 ± 1.39) days vs. (7.40 ± 1.10) days, *P* < 0.001], with an average difference of −3.1 days between the two groups, and a 95% confidence interval for the difference ranging from − 3.4 to −2.8 days (*P* < 0.001); Postoperative 24-hour VAS score [(2.76 ± 0.70) vs. (6.40 ± 1.10), *P* < 0.001], with an average difference of −3.3 points between the two groups, and a 95% confidence interval for the difference of −3.6 to −2.9 points (*P* < 0.001); Time to first oral intake [(2.19 ± 0.40) days vs. (3.85 ± 1.42) days, *P* < 0.001], with an average difference of −1.4 days between the two groups, and a 95% confidence interval for the difference of −2.1 to −0.8 days (*P* < 0.001); This indicates that we have 95% confidence that the advantage in the above indicators is attributable to the TOES + ENS technique. (See Fig. 6)

### Clinically meaningful metrics

**Fig. 7:**
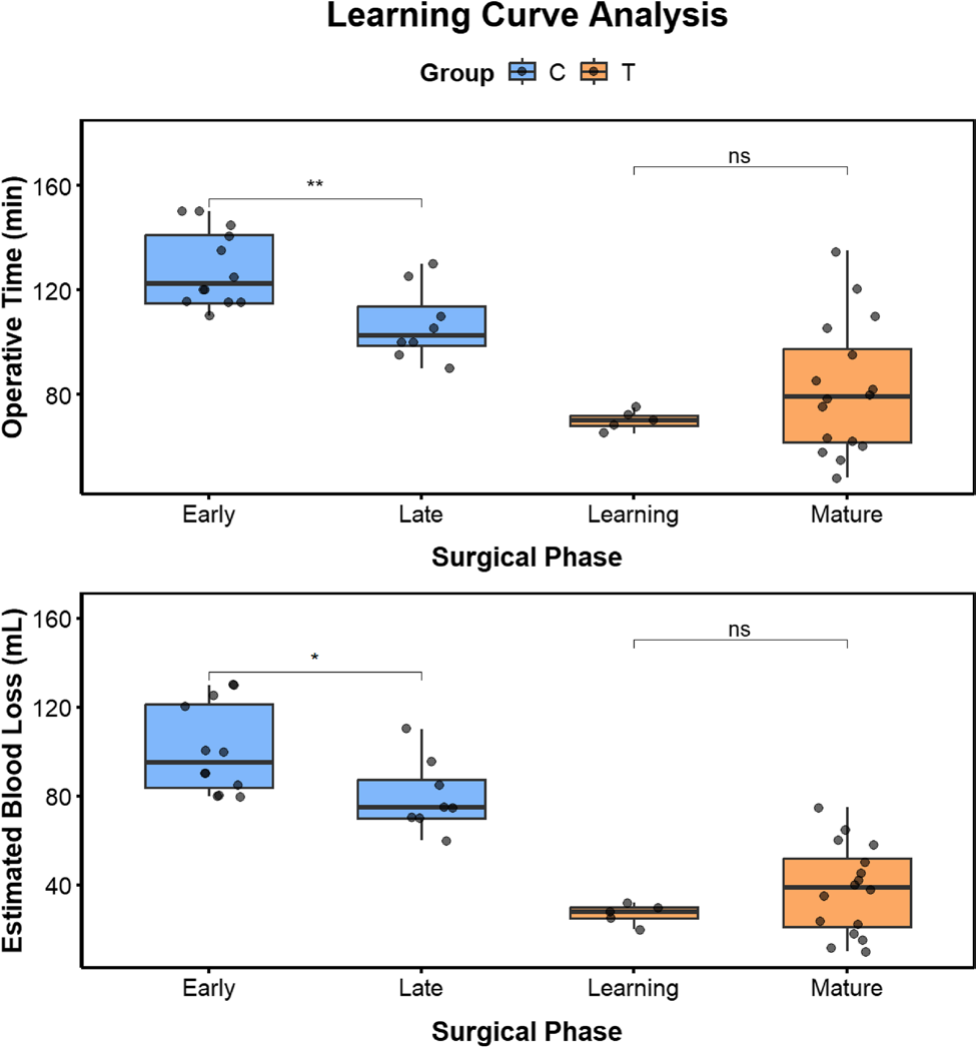
Learning Curve Charts for Group T and Group C

Compared with Group C, Group T had a Grade A wound healing rate of 95.2% vs. 70.0%, *P* = 0.45. After strict Bonferroni correction for multiple comparisons, the difference did not reach statistical significance after Bonferroni correction. The incidence of T-group nerve injury (4.8% vs. 20.0%, *P* = 0.311) did not reach statistical significance. Post-hoc power analysis for this key clinical endpoint indicated that the study’s power was only 35.1% at the current sample size, suggesting a high risk of Type II error. No significant differences were observed in terms of vascular injury (0 cases vs. 1 case), surgical cavity effusion/infection (1 case vs. 3 cases), or functional impairment rate (0 cases vs. 2 cases). (As shown in Fig. 7) 

### Subgroup analysis results

In subgroup analyses based on tumor location (anterior styloid process interval/posterior styloid process interval), the T-anterior styloid process interval group compared to the C-anterior styloid process interval group showed significant differences in surgical time (77.75 ± 18.94 min vs. 116.25 ± 15.43 min, *P* < 0.001), blood loss (34.00 ± 14.52 ml vs. 87.50 ± 15.34 ml, *P* < 0.001), and the T-posterior stem gap group had advantages over the C-posterior stem gap group in terms of surgical time (80.89 ± 25.31 min vs. 125.00 ± 18.71 min, *P* < 0.001) and blood loss (37.33 ± 21.11 ml vs. 100.00 ± 24.37 ml, *P* < 0.001). Intragroup analysis of the T group showed no significant statistical differences in surgical time (77.75 ± 18.94 min vs. 80.89 ± 25.31 min, *P* = 0.751) and blood loss (34.00 ± 14.52 vs. 37.33 ± 21.11 ml, *P* = 0.673).

In the subgroup analysis based on the surgical approach: relative to group C-TOES surgical approach without ENS (5 cases), the same advantages in terms of time (79.10 ± 22.49 min vs. 114.00 ± 14.63 min, *P*<0.001), bleeding volume (35.43 ± 18.16 ml vs. 86.00 ± 13.19 ml, *P*<0.001) were found Compared with group C-transcervical approach (15 cases), the operation time (79.10 ± 22.49 min vs. 121.67 ± 17.76 min, *P*<0.001) and bleeding volume (35.43 ± 18.16 ml vs. 94.67 ± 21.87 ml, *P*<0.001) were also superior.

### ANCOVA result

Additionally, we found that the SMD for “tumor diameter” between the two groups was > 0.2 (3.55 ± 1.42 cm vs. 4.06 ± 0.94 cm, SMD = 0.42, *P* = 0.187), suggesting a potential baseline imbalance. To adjust for differences in tumor diameter between groups, we performed an ANCOVA on surgery-related indicators (surgery time, blood loss) for both groups. The results showed that after controlling for the covariate “tumor diameter,” the study group (T group vs. C group) had a highly statistically significant effect on the outcome variable (surgical time) (F(1, 38) = 91.70, *p* < 0.001). Specifically, the adjusted mean value for the C group (adjusted surgery time = 116, SE = 2.42) was significantly higher than that for the T group (adjusted surgery time = 83, SE = 2.36). The 95% confidence intervals for Groups C and T were [110.8, 120.6] and [78.2, 87.7], respectively, with no overlap between the two. The study group assignment (T group vs. C group) also had a highly statistically significant effect on the outcome variable (bleeding volume) (F(1, 38) = 356.36, *p* < 0.001); The mean value for the C group (adjusted bleeding volume = 88.3, SE = 1.83) was significantly higher than that for the T group (adjusted bleeding volume = 39.4, SE = 1.79), with 95% confidence intervals of [84.6, 92.1] and [35.8, 43.0], respectively, showing no overlap between the two groups.

The results further confirm the significance and stability of the intergroup differences, indicating that the advantage of the TOES + ENS technique in this patient population is not attributable to differences in tumor diameter.

A notable finding in this study is that, in our multivariate model, tumor location (anterior to the styloid process vs. posterior to the styloid process) in the T group did not show the expected significant association with surgical time (*p* = 0.751). Given the more complex anatomical structure of the retrostyloid space, this result appears to contradict common clinical experience. We therefore propose a cautious hypothesis: the TOES + ENS surgical approach may offer certain specific advantages over traditional approaches when addressing anatomically complex regions. This technique utilizes endoscopy to provide surgeons with a more direct and close-up surgical field of view, particularly during deep manipulations. This may facilitate more efficient identification and protection of important neurovascular structures, thereby potentially reducing the surgical difficulty differences associated with different anatomical locations to some extent. This finding suggests that the value of the TOES + ENS technique may not only lie in the overall reduction of surgical time but also in its potential ability to address specific anatomical challenges.

### Learning curve analysis

Except for the T group, all other groups (C group) exhibited a significant learning curve effect. Compared with the Late cohort (*n* = 8), the Early cohort (*n* = 12) had significantly longer surgical times (128.33 ± 14.19 min vs. 106.88 ± 13.21 min, *P* = 0.003) and significantly increased intraoperative blood loss (100.83 ± 19.24 ml vs. 80.00 ± 15.00 ml, *P* = 0.014), indicating that the accumulation of surgical experience effectively improved the efficiency of traditional surgery.

In contrast, no typical decline in the learning curve was observed in the T group. There were no significant differences in surgical time (70.00 ± 3.41 min vs. 81.94 ± 24.39 min, *P* = 0.073) or intraoperative blood loss (27.00 ± 4.20 ml vs. 38.06 ± 19.44 ml, *P* = 0.048). Notably, in the “Mature phase,” the standard deviations for surgical time and blood loss significantly increased (surgical time standard deviation: 3.41 vs. 24.39; blood loss standard deviation: 4.20 vs. 19.44), which may suggest that as surgeons gain technical confidence, they tend to apply the technique to cases with more complex anatomical structures.

**Fig. 8:**
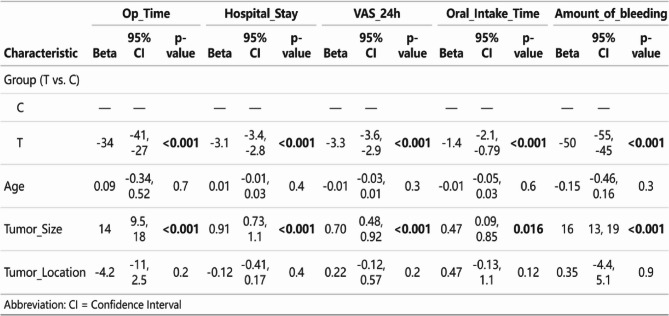
Multivariate regression analysis

Key cross-sectional comparisons showed that even in the “Learning phase” of the T group, surgical time and intraoperative blood loss were significantly superior to those of the “Late cohort” in the traditional surgery group (all indicators *P* < 0.001). After entering the “Mature phase,” the advantages of the T group over the Late cohort of the C group became even more pronounced (*P* < 0.001). These results strongly suggest that the superiority of TOES + ENS in terms of indicators (surgery time and blood loss) stems from the technique itself, rather than biases related to time or accumulated experience (as shown in Fig. 8).

### Sensitivity analysis and multivariate regression analysis

Sensitivity analysis showed that after excluding one case of low-grade malignant tumor in the T group, all major statistical conclusions of this study remained unchanged (surgery time, blood loss, 24-hour VAS postoperatively, number of days to first oral intake, length of hospital stay; *P* < 0.001). Additionally, this patient did not experience any postoperative complications, suggesting that the results of this study are not sensitive to the pathological type of a single case, and the conclusions are highly robust.

The results of the multivariate regression analysis showed that, after adjusting for age, tumor diameter, and location: (1) Surgery time: Compared with Group C, Group T was an independent predictor of shorter surgery time (Beta = −34, 95% CI: −41 to −27; *p* < 0.001), with an average reduction of 34 min. (2) Length of hospital stay: The T group was independently associated with shorter length of hospital stay (Beta = −3.1, 95% CI: −3.4 to −2.8; *p* < 0.001), with an average reduction of 3.1 days. (3) Postoperative 24-hour VAS: The T group was an independent predictor of lower postoperative 24-hour VAS scores (Beta = −3.3, 95% CI: −3.6 to −2.9; *p* < 0.001), with an average reduction of 3.1 days. (4) Shortest number of days to resume oral intake: The T group significantly predicted an earlier resumption of oral intake (Beta = −1.4, 95% CI: −2.1 to −0.79; *p* < 0.001), with an average reduction of 1.4 days. (5) Intraoperative bleeding: The T group was also a strong independent predictor of reduced intraoperative blood loss (Beta = −50, 95% CI: −55 to −45; *p* < 0.001), with an average reduction of 50 ml.

**Fig. 9:**
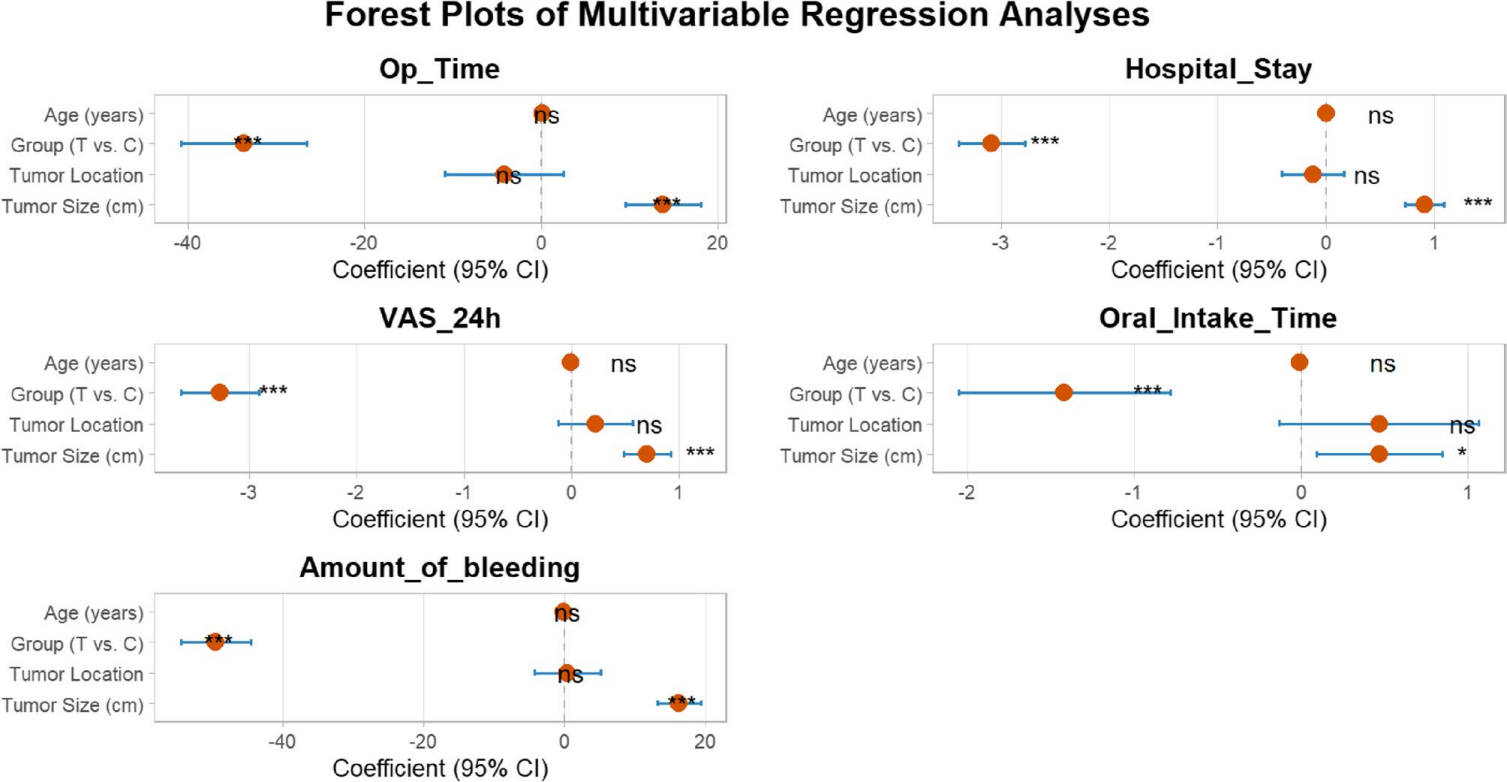
Forest plots for multiple regression analysis

**Fig. 10:**
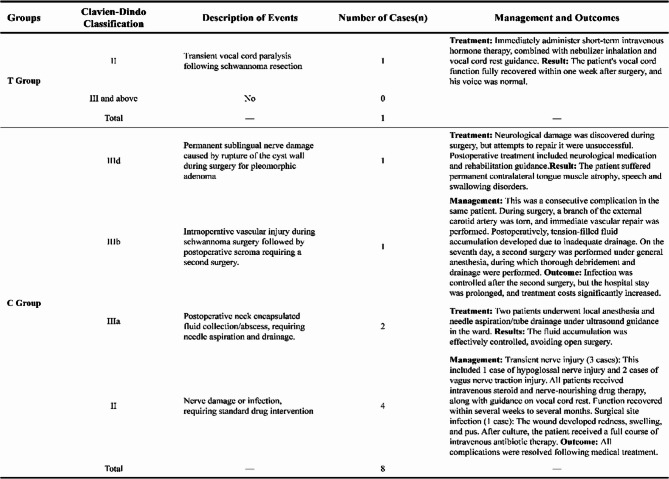
Postoperative Complications According to the Clavien-Dindo Classification

Additionally, in all models, tumor diameter was another important independent factor predicting worsening of clinical outcomes (e.g., prolonged surgery time, increased bleeding volume, etc.) (p-values ranging from < 0.001 to 0.016). In contrast, age and tumor location were not significantly associated with any outcome measures after adjusting for other factors. These results suggest that the superiority of the TOES + ENS technique is a robust finding independent of patient and tumor characteristics (as shown in Figs. 9 and 10).

### Long-term follow-up analysis

**Fig. 11:**
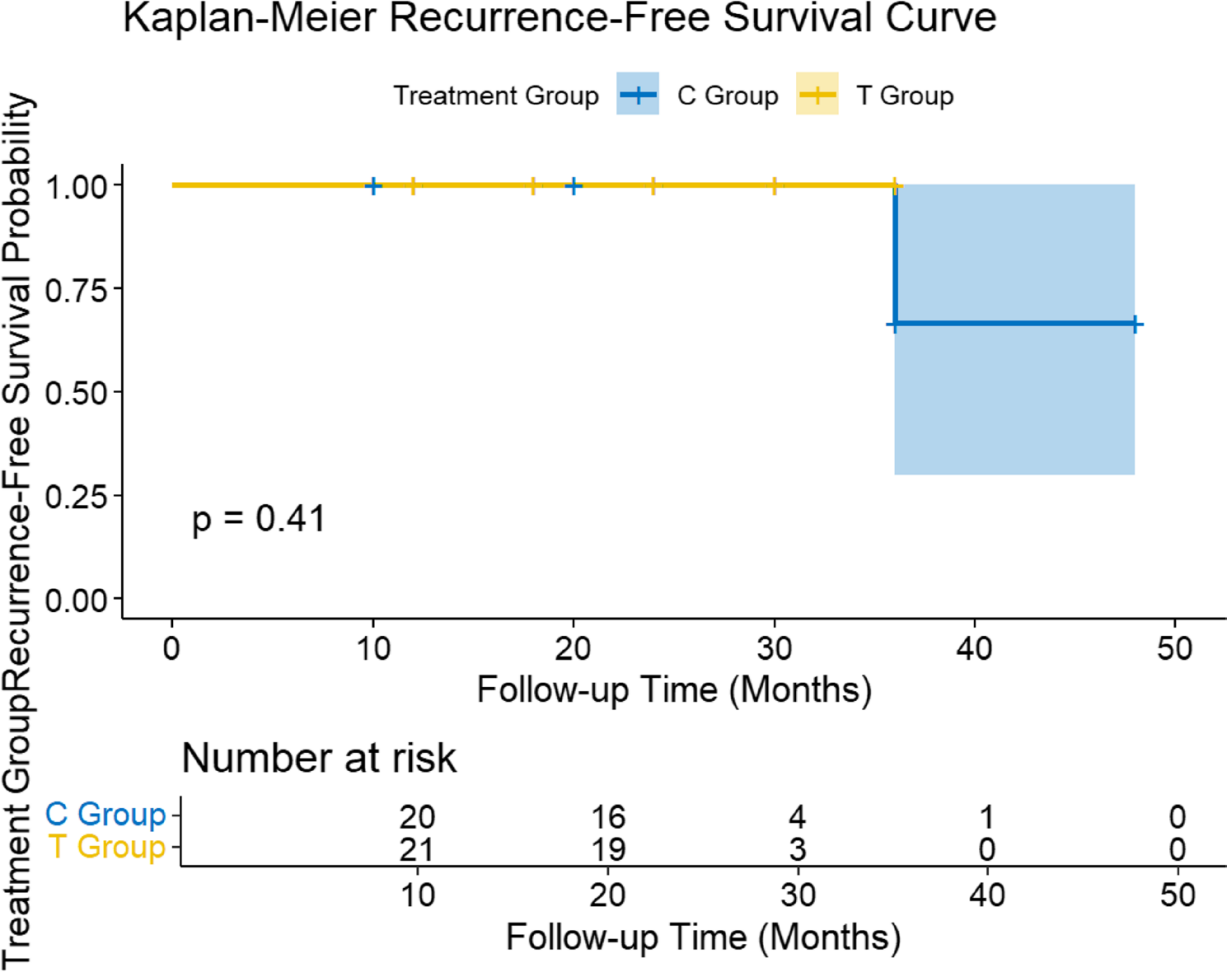
Kaplan-Meier Recurrence-Free Survival Curve

A total of 41 patients were included in the final long-term follow-up analysis (range: 1–48 months). The median follow-up time for both the T group and the C group was 24.0 months. During the follow-up period, one patient in the C group experienced tumor recurrence, while no patients in the T group experienced recurrence. Figure 11 shows the Kaplan-Meier recurrence-free survival curves for the two groups. Based on the curve estimates, the 1-year and 3-year recurrence-free survival rates for the TOES + ENS group were 100% and 100%, respectively, while the 1-year and 3-year recurrence-free survival rates for the traditional surgery group were also 100%. The log-rank test results showed that the difference in recurrence-free survival curves between the two groups was not statistically significant (*p* = 0.41) (as shown in Fig. 11).

## Discussion

### Technical advantages and clinical value of TOES + ENS

TOES + ENS technology combines preoperative imaging with real-time spatial localization technology, integrated with endoscopic visualization, to achieve precise resection of parapharyngeal space tumors. Our data show that the complete tumor resection rate (100%) in the T group is higher than the 89% reported for traditional transoral surgery, while the carotid artery injury rate (0%) is lower than the 7.3% reported for transneck surgery [9, 10]. The severity of complications is lower than in the C group, and the T group also demonstrates significantly superior outcomes in terms of surgical time, intraoperative blood loss, hospital stay, postoperative 24-hour VAS, and time to first oral intake compared to the C group. These “advantageous differences” are primarily attributed to the real-time tracking functionality of the ENS system—by fusing preoperative imaging with real-time anatomical structures during surgery, it precisely defines the spatial boundaries between the tumor and critical structures (such as the ICA), thereby avoiding the inherent risks of blind dissection in traditional surgery [11]. The safety of this surgical system is manifested in two aspects. First, in terms of spatial localization, the ENS navigation system ensures that the separation procedure is maintained at a safe distance of ≤ 2.5 mm from Important anatomical structures throughout (e.g., ICA), potentially offering neurofunctional protection [12]. Second, in terms of tissue manipulation, the use of a plasma knife replaces traditional blunt separation, with its low-temperature cutting and immediate hemostasis properties effectively avoiding the risks of microvascular tearing and rupture commonly associated with the latter [13]. In terms of wound healing quality, the T group demonstrated a clinically significant advantage, with an A-grade healing rate of 95.2%, higher than the 70.0% in the C group [*P* = 0.45; although the difference was not statistically significant after Bonferroni correction (*n* = 5, α’=0.01), it suggests a potential trend toward superiority in this metric]. Therefore, we believe that this result may stem from the refined surgical cavity management enabled by ENS. Specifically, ENS-guided pre-set negative pressure drainage minimizes dead space, while the endoscopic tension-free suturing and efficient hemostasis techniques assisted by ENS can reduce the core risk of incision infection, thereby directly promoting better healing outcomes. The core advantage of the TOES + ENS system lies in its ability to proactively identify and mitigate complications that are difficult to predict in traditional surgical procedures. In the context of oncology, the 5% incomplete resection rate in Group C is not merely a statistical figure; it ultimately manifested as a single case of long-term recurrence due to capsule rupture and tumor residue. Our approach employs a dual-insurance strategy of “preoperative 3D planning + intraoperative real-time monitoring,” achieving a 100% complete resection rate and eliminating the seeds of recurrence from a methodological standpoint. From an anatomical perspective, the Grade IIIb postoperative complication in Group C, caused by damage to the external carotid artery branch, exposed the shortcomings of traditional surgery in protecting delicate anatomical structures. This further underscores the unique value of ENS: it is not merely a passive avoidance technique but an active “vascular mapping” tool [14]. By pre-marking high-risk vascular pathways and enabling precise endoscopic microvascular techniques, it ensures high-quality postoperative healing.

### Potential of TOES + ENS technology in dealing with complex anatomical regions

One notable finding in this study that warrants further exploration is that our subgroup analysis based on tumor location preliminarily suggests that the advantages of the TOES + ENS technique may be more pronounced in managing tumors in the more anatomically challenging retrostyloid space. However, when tumor location was included as a covariate in the multivariate regression model, its independent predictive role for various clinical outcomes did not reach statistical significance. This phenomenon may be attributed to multifactorial causes. First, tumors in the retrosigmoid space often present greater surgical challenges, and their influence may have been diluted by stronger predictive factors in the model, such as tumor diameter or the surgical approach itself. Second, as a single-center, exploratory study with a limited sample size, this research may lack the statistical power to identify the relatively weak independent effect of “location” in a complex multivariate model.

Therefore, we cautiously interpret this as follows: subgroup analysis may have revealed a clinically significant trend, indicating that the TOES + ENS technique holds great potential in addressing complex anatomical regions; however, the results of the multivariate regression remind us that, at the current level of evidence, surgical technique selection and tumor volume remain important factors influencing prognosis. Exploring the compatibility between tumor subregions (such as the posterior styloid process) and different minimally invasive techniques will be a highly valuable direction for future research.

### Learning analysis of TOES + ENS technology

This study, spanning seven years, raises questions about the potential confounding effects of learning curve effects and technological evolution. We did not avoid this issue but instead conducted a thorough investigation through detailed subgroup analysis. The analysis revealed a dual phenomenon: on one hand, Group C did exhibit experience-dependent performance, with efficacy improving over time; on the other hand, the TOES + ENS technique demonstrated the potential to surpass traditional surgery even in its initial application phase.

A more significant finding was that, during the mature application phase of the TOES + ENS technique, key surgical indicators tended toward stability rather than further improvement, but the variability of outcomes increased. We boldly infer that surgeons are not merely repeating simple procedures but are expanding the application boundaries of the technique to address more complex tumors. This uniquely validates the powerful functionality and safety of the technical platform. Therefore, this study, through quantitative analysis of the learning curve, not only effectively eliminates the interference of time bias but further highlights the independent and robust technical advantages of the TOES + ENS technique in managing parapharyngeal space tumors. A more significant finding is that, unlike the significant experience dependency observed in Group C, the TOES + ENS technique did not exhibit the typical steep learning curve. Even during its “learning phase” at our center, its performance in terms of surgical time and bleeding control already surpassed that of traditional surgery in its mature phase. This phenomenon may suggest that the superiority of the TOES + ENS technique primarily stems from its technical platform itself, rather than being highly dependent on the surgeon’s long-term experience accumulation. This suggests that the technology may have a flatter learning curve and lower training threshold, potentially conferring unique advantages in terms of standardized promotion and widespread adoption compared to other complex minimally invasive techniques in the future.

### Implementation framework: training requirements, and a proposed patient selection algorithm

**Fig. 12:**
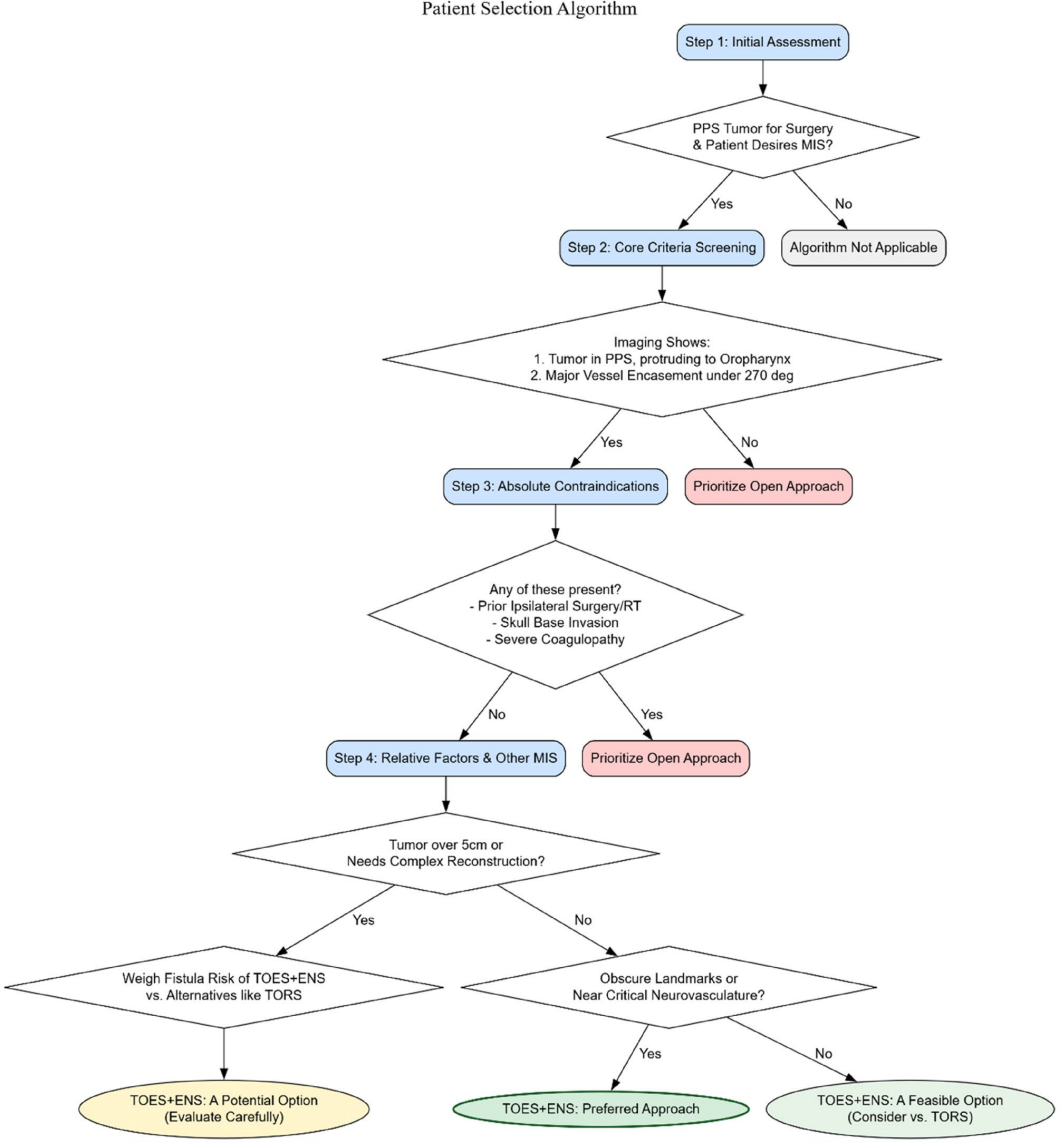
Patient selection algorithm

Based on our research and previous experience, we propose the following stepwise patient selection algorithm to guide clinical decision-making (flowchart): Step 1: Initial assessment. Identify parapharyngeal space tumors requiring surgical resection, and confirm that the patient has minimal surgical intent. Step 2: Core criteria screening. Imaging demonstrates that the tumor mass is located in the parapharyngeal space, protruding into the oropharynx, and has not encased important structures such as the internal carotid artery by more than 270 degrees. Step 3: Exclusion of absolute contraindications. Assess whether any of the following conditions are present: prior surgery or radiotherapy on the same side, extensive tumor invasion of the skull base, severe coagulation disorders, etc. (see the exclusion criteria in the “Methods” section for details). If any of these conditions are present, traditional open surgery should be prioritized. Step 4: Weigh relative factors against other minimally invasive options. If there are no absolute contraindications, proceed to detailed decision-making. If the tumor is large (> 5 cm) or complex reconstruction is required postoperatively, carefully assess the risk of pharyngeal fistula with TOES + ENS and compare it with techniques such as TORS. The advantage of TOES + ENS lies in its ability to manage cases with unclear anatomical landmarks or tumors adjacent to vessels and nerves (e.g., Fig. 12).

### Follow-up outcome

In this study, although some secondary endpoints showed advantages for the T group, from the perspective of long-term tumor control, we found through Kaplan-Meier survival analysis that there was no statistically significant difference in recurrence-free survival rates between the two surgical approaches. This suggests that, in terms of long-term efficacy, the TOES + ENS technique is at least non-inferior to traditional surgical procedures in terms of tumor eradication efficacy.

### Meta-analysis and systematic review

The results of this study should be interpreted within the context of the evolving trend toward minimally invasive surgery for parapharyngeal space tumors. Although various minimally invasive techniques have continued to advance over time, the treatment of parapharyngeal space tumors remains challenging when tumors lack clear anatomical landmarks or are closely associated with important structures such as the internal carotid artery.

In recent years, various minimally invasive techniques have emerged, aiming to reduce the trauma associated with traditional open surgery. It is widely recognized that minimally invasive approaches represent an important direction for the future of parapharyngeal space surgery. Recent systematic reviews also support the notion that minimally invasive techniques are associated with better perioperative outcomes compared to traditional open surgery [15]. However, these studies also suggest that when tumors are adjacent to anatomical structures such as the ICA or skull base, how to ensure functional preservation while maintaining thorough resection remains a concern in clinical practice [16]. The original intention of this study was to explore whether the introduction of ENS could help address this challenge.

One of the most striking findings of this study is that the TOES + ENS technique achieved a 100% microscopic complete resection rate (R0) in our cohort. This result is clinically significant because even large medical centers have not been able to completely avoid the risks of incomplete resection and postoperative recurrence, as reported in the literature. For example, a large-scale study involving 175 surgical patients reported that the transcervical approach was the most preferred surgical method for 131 (75%) cases, while the transcervical-parotid and transoral approaches were used for 11 (6%) and 20 (11%) cases, respectively. Other approaches included the transnasal, mandibular split, and combined approaches), a recurrence rate of 1.7% was still observed postoperatively [17]. Another study spanning 20 years of experience showed that in traditional open surgery primarily using the cervical-transparotid approach, the macroscopic complete resection rate was only 86%, and in some cases, complete resection was intentionally abandoned to preserve nerve function [18].

We believe that the fundamental reason traditional surgery cannot achieve a 100% R0 resection rate lies in its limited deep visualization and lack of three-dimensional spatial perception, which increases the risk of capsule rupture in high-risk areas. The results of this study suggest that ENS technology effectively addresses this core deficiency. By providing real-time, precise information on the tip position of the instrument within 2.5 mm, the navigation system constructs a safe “macroscopic surgical corridor” for the surgeon, significantly enhancing confidence and certainty in operating within confined spaces. This allows the surgeon to perform dissection along the tumor capsule more confidently and precisely under endoscopic magnification, thereby maximizing the avoidance of capsule rupture and tumor residue. Therefore, the 100% R0 resection rate we achieved is not coincidental but a direct reflection of how this technological platform fundamentally addresses the inherent challenges of traditional surgery.

The complication profile in this study contrasts significantly with the inherent risks of traditional open surgery documented in recent meta-analyses. In our T-group cohort (21 cases, primarily composed of pleomorphic adenomas and schwannomas), only one transient neurological event was recorded (temporary nerve dysfunction following schwannoma resection). This finding is particularly notable when compared with the conclusions of Faisal et al. (2023). Their meta-analysis found that the incidence of neurological complications from traditional open surgery for neurogenic tumors reached 48%, with vocal cord paralysis identified as the primary disabling factor [19]. We infer that while TOES provides an enlarged field of view, it is the real-time three-dimensional guidance provided by ENS that enables surgeons to confidently dissect along the delicate capsule of the schwannoma, thereby reducing the incidence of nerve injury.

The perioperative outcomes in this study not only align with the benchmark established by recent meta-analysis data for endoscopic-assisted transoral approach (EATA) but appear to surpass it. Chen et al. (2021) strongly confirmed the superiority of EATA over traditional open approaches in their meta-analysis, with an average difference in surgical time of approximately 5.56 min in the EATA group, intraoperative blood loss was approximately 89.02 ml, and the mean difference in hospital stay was 2.44 days. In direct comparison, the mean difference in surgical time in the T group of this study was approximately 34.00 min, intraoperative blood loss was approximately 50.00 ml, and the mean difference in hospital stay was 3.10 days. Additionally, five independent studies included in the analysis showed that the average surgical time in the EATA cohort ranged from 57.7 to 127 min, with four of these studies reporting average surgical times longer than those in the T group of this study [20]. 

This direct data comparison reveals a further incremental improvement. Previous literature, as persuasively summarized by Chen et al., has established that endoscopic transoral surgery is a less traumatic and more efficient alternative to open surgery; our findings, however, present a new argument: the integration of ENS technology and TOES appears to elevate this modern technique to a higher performance tier. By providing unparalleled surgical certainty, ENS likely minimizes the time spent on anatomical dissection and reduces unnecessary tissue damage, thereby collectively contributing to a faster and clearer surgical field.

Portable, user-friendly ENS is a significant recent trend. A notable example is the work of Lang et al. (2025), which represents a major technical breakthrough in the field in terms of portability and usability. They successfully developed a new portable neuro-navigation system and, importantly, established an extremely high precision benchmark: achieving a TRE < 2.0 mm high-precision standard in laboratory models [21]. 

However, the highest TRE accuracy was achieved under idealized experimental conditions, and its non-comparative design prevented it from directly quantifying the clinical benefits of this technical accuracy. In contrast, our research focuses on validating clinical efficacy in real-world settings. We validated the clinical benefits of TRE < 2.5 mm in the more challenging setting of oral and maxillofacial surgery and, through our comparative study design, strongly demonstrated that this level of precision is not only clinically sufficient but also effective. We quantitatively confirmed that this real-world precision can directly translate into superior clinical outcomes—including a 100% R0 resection rate, significantly reduced complications, and significantly advantageous perioperative indicators. This provides strong evidence that, for this special and complex anatomical region, the primary value of ENS lies in providing a definitive “macro surgical corridor” that fundamentally enhances surgical safety and efficacy.

The results of this study are consistent with the aforementioned trends. Additionally, this study not only reaffirms the long-term oncological safety of TOES technology in the treatment of parapharyngeal space tumors—its 36-month recurrence-free follow-up results align with those of existing multicenter studies [22]—but also retains the unparalleled aesthetic advantages and neural function preservation (particularly avoiding the risk of mandibular nerve injury) inherent in the natural orifice approach, which holds decisive appeal for younger patient populations. However, we innovatively integrated ENS seamlessly into the TOES workflow, achieving real-time fusion of preoperative 3D modeling and intraoperative navigation to control tumor margin identification errors within 2.5 mm.

Among current minimally invasive techniques, TORS is an important one, characterized by its three-dimensional visualization and operational flexibility, particularly offering advantages in cases requiring precise suture reconstruction [23]. However, TORS operations still primarily rely on the surgeon’s direct visual judgment. When anatomical landmarks are unclear, there remains a risk of damaging surrounding structures. The core advantage of the TOES + ENS combined approach explored in this study lies in its precise “mapping” capability. By providing real-time feedback on anatomical position information during surgery, it offers surgeons a “transparent” field of view beyond the capabilities of the naked eye and conventional endoscopes, making it particularly suitable for tumors with unclear borders adjacent to important blood vessels and nerves. This complements the dexterous manipulation of TORS in functional terms rather than simply competing with it. Other approaches (such as endoscope-assisted trans-neck approaches) can also achieve minimally invasive surgery, but when dealing with tumors in higher locations, their exposure angle and operational depth may be limited [24].

### Alternatives, limitations, and contraindications

Although the prospects for TOES + ENS technology are highly promising, it is not without limitations and contraindications. First, the lack of prospective “head-to-head” comparisons with other minimally invasive techniques, such as TORS, is a major limitation of this study, and future multicenter randomized controlled trials will be necessary. Second, the technique has relative contraindications. Absolute contraindications include: (1) severe mouth opening restriction (e.g., less than 3.0 cm); (2) Imaging studies indicate that the tumor has extensively invaded the carotid sheath or cranial base bone. Relative contraindications include: 1) Tumor diameter is too large (e.g., > 5 cm), which may result in excessive pharyngeal wall defects postoperatively, increasing the risk of pharyngeal fistula (although both groups included cases with large-volume [> 5 cm] parapharyngeal space tumors, and the surgical methods in both groups were sufficient for resection. However, for Group C, the technical feasibility of this approach often comes at the cost of sacrificing a large amount of normal tissue to achieve sufficient surgical exposure, resulting in a higher likelihood of postoperative complications compared to cases < 5.0 cm. The core objective of this study is to investigate whether the TOES + ENS technique can serve as a less invasive, functionally superior alternative for challenging tumors of equivalent volume.

Our data provide a definitive answer. Although both groups included tumors exceeding 5 cm in diameter, the TOES + ENS technique, leveraging the millimeter-level precision enabled by navigation, allowed for more precise dissection of tumor margins. This precision directly translates into the maximum preservation of adjacent healthy parapharyngeal tissues and mucosa, thereby fundamentally reducing the risk of complications caused by extensive resection—a case of a patient with a 6.8 cm tumor in the T group who experienced only minimal postoperative fluid accumulation, achieved Grade A healing, and had no pharyngeal fistula, serves as a compelling example. Therefore, the value of TOES + ENS technology lies not merely in expanding the upper limit of surgical size but in providing a new paradigm for the treatment of large tumors that prioritizes both safety and functionality. 2) Patients who have previously undergone neck radiotherapy may experience tissue adhesion and unclear anatomical layers, which can affect the accuracy of the navigation model. Clinicians must comprehensively assess the patient’s individual condition and tumor characteristics when selecting a surgical plan to make the most reasonable decision.

### Limitations and future directions

One of the most significant limitations of this study is the relatively small sample size, which directly impacts the statistical power of between-group comparisons for low-incidence but clinically important complications. Specifically, we observed that the rate of nerve injury in the T group (4.8%) was significantly lower than that in the C group (20.0%), a difference of clinical significance. However, due to insufficient statistical power (post-hoc power = 35.1%), this difference did not reach statistical significance. This implies that our study conclusions have a substantial likelihood (65%) of underestimating the safety advantage of the new technology, i.e., a Type II error. To provide guidance for future study designs, we conducted a prospective sample size estimation. The results indicate that to validate the observed magnitude of difference in nerve injury rates with 80% power at a significance level of α = 0.05, future studies would require approximately 124 patients (62 per group). Therefore, the findings regarding neural function protection in this study should be cautiously interpreted as an important, hypothesis-generating finding rather than a definitive conclusion. The precise efficacy of the technology awaits further confirmation through future large-scale, multicenter, prospective randomized controlled trials.

The finding that there was no significant association between tumor location and surgical timing should also be considered in light of other possible explanations. The sample size of this study may have limited our statistical power to detect smaller effect sizes. Additionally, as a retrospective study, we cannot completely rule out the possibility of selection bias, such as more experienced surgeons being assigned more challenging cases. Therefore, future prospective studies with larger sample sizes are needed to further validate and interpret this finding.

Another significant limitation of this study lies in the heterogeneity within Group C, which includes both open trans cervical approaches and TOES without ENS. We did not perform a stratified analysis to compare the TOES + ENS technique separately with these two traditional techniques. This combined analysis may obscure or dilute the true effect size of the TOES + ENS technique compared to each individual approach. For example, if compared solely with the open trans-cervical approach, the advantages observed in this study regarding reduced blood loss and hospital stay duration might be more pronounced. Future studies should ideally adopt a prospective, multi-arm trial design to elucidate these subtle differences and conduct independent “head-to-head” comparisons between the TOES + ENS technique, TORS technique, and each traditional approach.

Additionally, the assessment of certain outcomes in this study relied on subjective measurements. First, regarding the evaluation of wound healing grades, to minimize assessor bias, all wound healing conditions were independently assessed by two senior otolaryngologists who were not involved in the specific surgeries, using a predefined standardized chart. When results were inconsistent, consensus was reached through discussion. Although we attempted to ensure consistency through this process, this study did not conduct formal statistical analyses of inter-rater consistency (e.g., calculating Cohen’s kappa coefficient), which constitutes a methodological limitation of this study.

Second, regarding the 24-hour VAS postoperatively, we used the internationally recognized Visual Analogue Scale for quantification. Although the VAS is a validated, standardized tool for assessing pain intensity, it is inherently a patient-reported outcome, and its results inevitably reflect the patient’s individual subjective experience and may be influenced by various factors such as personal pain thresholds and anxiety levels. Therefore, when interpreting this data, its inherent subjectivity should be taken into account. Finally, when evaluating semi-subjective indicators such as postoperative complications, we strictly adhered to internationally recognized standardized grading systems (e.g., the Clavien-Dindo classification), providing a unified and clear standard for assessment. This minimized the arbitrariness of individual judgments and ensured the standardization and consistency of the evaluation process.

Despite these efforts, it is impossible to completely eliminate all possibilities of measurement bias. Therefore, the findings of this study should be regarded as an important preliminary exploration of the efficacy and safety of the TOES + ENS technique. The final confirmation of these conclusions will require future, more rigorously designed prospective, single-blind, or even double-blind randomized controlled trials for further validation.

Another limitation of the study is the failure to explore the quantitative relationship between navigation technology performance parameters and clinical outcomes. Although we confirmed that the registration error of the navigation system met high-precision standards (e.g., ≤ 2.5 mm) in each case, these data were used as single-use surgical quality control standards rather than systematically collected and continuously monitored as study variables. Therefore, we were unable to analyze the correlation between technical indicators such as initial registration error or intraoperative accuracy drift and clinical outcomes such as surgical time and blood loss. Future prospective studies should focus on establishing a standardized data collection process to systematically assess the impact of these technical parameters, thereby gaining a deeper understanding of the underlying mechanisms underlying the benefits of navigation technology.

Regarding tumor heterogeneity, although the patient cohort in this study included one case of malignant tumor, after conducting an in-depth analysis of the specific circumstances of this case, we concluded that it did not pose a substantial threat to the validity of the study’s conclusions.

First, from the perspective of surgical technique execution, this case of low-grade malignant BCAC presented on imaging as a well-defined mass with a complete capsule-like structure. This characteristic aligns the core surgical challenge with that of the majority of benign lesions in this study, such as pleomorphic adenomas: achieving “en-bloc resection” of the tumor while ensuring absolute integrity of the capsule. This differs fundamentally from the principle of performing prophylactic wide local excision to obtain several centimeters of safety margins when treating high-grade malignant tumors. Therefore, despite differing pathological diagnoses, this case shares high technical homogeneity with other cases in the group in terms of surgical objectives, anatomical layer identification, and resection methods.

Second, the design of this study was intended to faithfully reflect the real-world clinical management of parapharyngeal space tumors. Our inclusion criteria were strictly based on the anatomical characteristics of the tumor (location, volume, adjacent relationships, etc.), rather than the pathological type, which is often unknown prior to surgery. In clinical practice, the choice of surgical approach is almost always made prior to the receipt of the final pathological report. Although we reported preoperative registration errors in the navigation system, we did not perform quantitative dynamic validation of navigation accuracy in the specific parapharyngeal space during surgery. Tissue displacement during surgery may result in discrepancies between actual accuracy and registration accuracy. To address this, our surgical strategy emphasizes the use of stable deep bony structures such as the pterygoid process and styloid process to continuously and dynamically anatomically cross-validate ENS information, ensuring surgical safety. Future research could explore integrating intraoperative imaging techniques (such as intraoperative CT or ultrasound) to more precisely quantify and correct such errors.

In addition, while the electromagnetic navigation technology employed in this study significantly improved surgical precision, its inherent technical limitations must also be carefully considered. First, the electromagnetic navigation system may be subject to potential interference from metallic instruments in the surgical field. Certain metallic surgical instruments used during the procedure (e.g., drills, suction tips) may interfere with the magnetic field, causing temporary shifts in navigation accuracy. To mitigate this issue, we used verified non-magnetic or weakly magnetic instruments during surgery and required that the tips of instruments maintain a safe distance from the navigation probe when operating near critical anatomical structures.

Second, the classic “brain-shift” issue [25] also exists in endoscopic sinus surgery, where tissue resection or morphological changes during surgery (such as tumor volume reduction or mucosal swelling resolution) can cause navigation registration failure. In this study, we repeatedly verified the accuracy of anatomical landmarks before and after resection of large lesions to assess the extent of registration offset. However, for cases with significant tissue deformation, the real-time accuracy of navigation may decrease to some extent.

Finally, maintaining stable patient-image registration throughout the entire surgery is another major challenge. Minor patient movement during surgery or accidental contact with the reference frame attached to the patient may cause registration failure. We minimize such risks by using a head frame for rigid head fixation and securely positioning the reference frame on the patient’s forehead, but eliminating the possibility is highly unlikely. These technical limitations are key areas for future iteration and optimization of navigation technology.

If we were to correlate preoperative capsule integrity with long-term recurrence rates, this would undoubtedly be a scientifically exciting endpoint. However, the extremely low number of recurrence events in our cohort renders meaningful statistical analysis infeasible. This remains a critical unresolved question. In the future, conducting larger-scale prospective studies is imperative to accumulate sufficient event numbers to statistically validate the hypothesis that “achieving better capsule preservation through techniques such as TOES + ENS directly translates into a lower risk of tumor recurrence.”

In addition, we primarily focused on perioperative safety and short-term efficacy, failing to systematically employ standardized assessment scales to conduct in-depth evaluations of patients’ long-term swallowing function, speech quality, and Quality of Life (QoL). Given the complexity of the anatomical structure of the parapharyngeal space and its impact on these critical functions, this undoubtedly represents an important area for future research to prioritize. We recommend that future prospective studies routinely incorporate validated assessment tools such as the MD Anderson Dysphagia Inventory (MDADI), the European Organization for Research and Treatment of Cancer Quality of Life Core Questionnaire (EORTC QLQ-C30), and its Head and Neck Module (H&N35) to more comprehensively demonstrate the true impact of this surgical procedure on patients’ long-term functional outcomes and quality of life.

Although this study shows the superiority of TOES + ENS technology in clinical outcomes, a comprehensive evaluation must acknowledge the economic costs and resource allocation issues involved in its implementation. We acknowledge that this study did not conduct a formal health economic analysis, which is a limitation; however, based on our data and previous literature, we can provide a preliminary outline of its cost-effectiveness profile.

First, the introduction of an electromagnetic navigation system requires significant capital investment and ongoing operational costs. The initial procurement cost of an ENS system can reach hundreds of thousands of dollars, in addition to annual maintenance agreements and ongoing expenses for essential single-use consumables (such as disposable probes and patient trackers). A cost analysis of emerging minimally invasive surgery (transoral endoscopic thyroidectomy) found that specialized instruments alone could increase direct costs by nearly $500 per procedure [26]. This aligns with our observation that TOES + ENS technology incurs higher direct material costs due to its high specificity.

However, these increased costs must be weighed against potential direct and indirect benefits. One of the core findings of this study is the significant reduction in surgical time. According to an authoritative analysis of financial data from hospitals in California, the average direct cost per minute in the operating room (OR) (including personnel salaries, consumables, etc.) is approximately $36 to $37, while the total cost (including indirect management and facility fees) is even higher [27]. Therefore, the time saved per procedure in this study can directly translate into thousands of dollars in cost savings. More importantly, we must recognize that the true economic value of this technology may lie more in avoiding high-cost complications. A cost-effectiveness model study of electromagnetic navigation bronchoscopy (ENB) noted that ENB is cost-effective largely because it reduces the incidence of adverse events such as pneumothorax associated with traditional needle biopsy and the subsequent treatment costs. Similarly [28], the medical expenses saved by avoiding any secondary interventions or long-term rehabilitation due to damage to critical structures through the enhanced surgical precision of TOES + ENS technology could be enormous, potentially far exceeding the premium cost of the technology itself [29]. 

Secondly, the successful deployment of TOES + ENS technology requires systematic infrastructure support and an understanding of “hidden costs.” This includes not only a modern operating room with sufficient space and seamless integration with imaging systems (PACS), but also clear technical support and maintenance agreements with suppliers to ensure stable operation and timely updates of the equipment. Human resource investment is equally critical. Xue et al.‘s study on the ENB learning curve provides valuable insights, indicating that surgeons typically require over 40 procedures to achieve technical proficiency and may initially prioritize simpler cases to ensure success rates during the learning phase [30]. This suggests that any center planning to adopt this technology must establish a multidisciplinary training system encompassing surgeons, nurses, and technical staff, and factor the time and resource costs associated with the learning curve phase into overall cost considerations.

Although this study demonstrates promising results of TOES combined with ENS technology in the treatment of parapharyngeal space tumors, we must clearly recognize its inherent limitations, which collectively define the scope of application of the study’s conclusions.

First, this study is essentially a single-center experience. This constitutes its primary limitation. The success of parapharyngeal space tumor surgery highly depends on advanced navigation devices, high-definition endoscopic systems, and a well-trained multidisciplinary team. Our study results reflect the best practices of our center under specific hardware and software configurations and team collaboration models. Therefore, the reproducibility of these results in other medical institutions with different equipment conditions or team experience requires further validation.

Second, the study results may be influenced by the specificity of the population and pathology. Our study population originated from a specific geographic region and may share certain commonalities in some aspects. More importantly, parapharyngeal space tumors exhibit diverse pathological types, with significant variations in size, texture, blood supply, and relationships with surrounding critical vascular and neural structures. The case selection in this study may not have fully encompassed all complex clinical scenarios, so the universality of this technique for tumors of different pathological types remains to be validated in a broader context.

Furthermore, as a highly precise, minimally invasive surgery performed in an anatomically complex region, the outcomes of this procedure are highly operator-dependent. Precise manipulation within the narrow three-dimensional space of the parapharyngeal space, which is densely populated with critical nerves and vessels, imposes extremely high demands on the surgeon’s learning curve. The results of this study are largely attributable to the surgical team’s long-term exploration and experience accumulation in this technical field. Therefore, for surgeons with limited experience or those in the early stages of the learning curve, directly replicating the superior data from this study may present challenges.

In summary, we believe this study should be regarded as an important early exploration of the application of TOES + ENS technology in the cutting-edge field of parapharyngeal space tumors. Its core value lies in providing preliminary evidence regarding the clinical feasibility, safety, and efficacy of this highly promising new technology. To overcome the aforementioned limitations, we strongly recommend and anticipate the conduct of large-scale, multi-center, prospective cohort studies or randomized controlled trials in the future, with the aim of providing higher-level evidence to support the safe and standardized application of this technology in broader clinical practice.

We fully recognize that to assess the ultimate value of this innovative surgical technique, patient feedback must be included. Therefore, in our future prospective multicenter studies, patient-reported outcomes (PROs) have been established as core secondary endpoints. We will utilize internationally recognized and validated scales, such as the University of Washington Quality of Life Scale (UW-QOL) or the European Organization for Research and Treatment of Cancer (EORTC) QLQ-H&N35 module, to systematically assess patients at multiple pre- and post-operative time points (e.g., 3 months, 1 year, 3 years) to comprehensively measure the true impact of the TOES + ENS technique on patients’ long-term quality of life, functional recovery, and aesthetic satisfaction. From a technical perspective, flexible endoscopic instruments equipped with force feedback systems can be developed to overcome the anatomical limitations of the “deep narrow space” of the parapharyngeal space—for example, flexible endoscopes combined with AI-assisted path planning can reduce the risk of capsule rupture [31]. Future research could also focus on integrating ENS with optical molecular imaging (such as methylene blue fluorescence) to enable real-time detection of minimally invasive tumor lesions [32].

Among the shortcomings of this study, the most important is that it is a single-center retrospective analysis with a limited total sample size. After stratifying the control group into a “TOES group” and a “Transcervical approach group” to improve methodological rigor, the sample size of each subgroup was further reduced. This inherently limited our statistical power to detect potential differences in some clinical outcomes. Therefore, some of the results that did not reach statistical significance still need to be further validated in future large-sample, multicenter prospective studies.

## Conclusion

For cases of parapharyngeal space tumors that strictly adhere to the inclusion criteria, the TOES + ENS technique demonstrates promising application prospects. Compared with non-navigated TOES and traditional trans-neck approaches, this study confirms that the combined technique is associated with lower intraoperative blood loss, lower 24-hour VAS scores postoperatively, shorter surgical duration, hospital stay, and time to first oral intake. In this study cohort, all patients in the T group achieved complete tumor resection (R0 resection rate of 100%) through real-time spatial localization, and the incidence of major complications remained at a low level (4.8%).

This study found that the advantages of this technique are particularly prominent in managing benign lesions with specific anatomical features. Its ideal indications include tumors located in the oropharynx that grow outward and do not invade the ICA or skull base (e.g., pleomorphic adenoma, schwannoma). Compared to non-navigated TOES and traditional trans-cervical approaches, ENS-guided extra-capsular resection is theoretically more precise.

However, the limitations of this technique must be emphasized. Complex lesions involving ICA encasement exceeding 270° or skull base invasion should be approached with caution. Additionally, the successful resection of a single case of BCAC included in this study should be strictly regarded as an exploratory observation. This individual case can only be used to generate preliminary hypotheses and is insufficient to confirm the oncological safety of this technique in malignant tumor treatment. The safety and efficacy of such applications require validation through dedicated long-term oncological studies.

In summary, the TOES + ENS technique is a safe and effective minimally invasive surgical option for treating benign parapharyngeal space tumors with suitable anatomical locations. Its ultimate clinical value remains to be confirmed by larger-scale prospective cohort studies. Future research directions should prioritize health economic analyses to assess cost-effectiveness and systematically incorporate PROs to comprehensively evaluate their impact on quality of life.

## Supplementary Information

Supplementary material 1.

Supplementary material 2.

## Data Availability

The datasets used and/or analysed during the current study are available from the corresponding author on reasonable request.
