# Supplementary figures and images for: Electromagnetic navigation-guided TOES for parapharyngeal tumors: a comparative study on precision and safety
Source: BMC Surg. 2025 Oct 24;25:500. doi: 10.1186/s12893-025-03206-y (PMC12553193; doi:10.1186/s12893-025-03206-y)

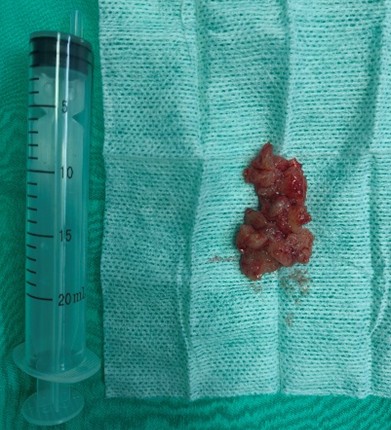

Supplement: Supplementary file 1 — Supplementary material 1. [file 12893_2025_3206_MOESM1_ESM.zip › Fig2-F.jpg]

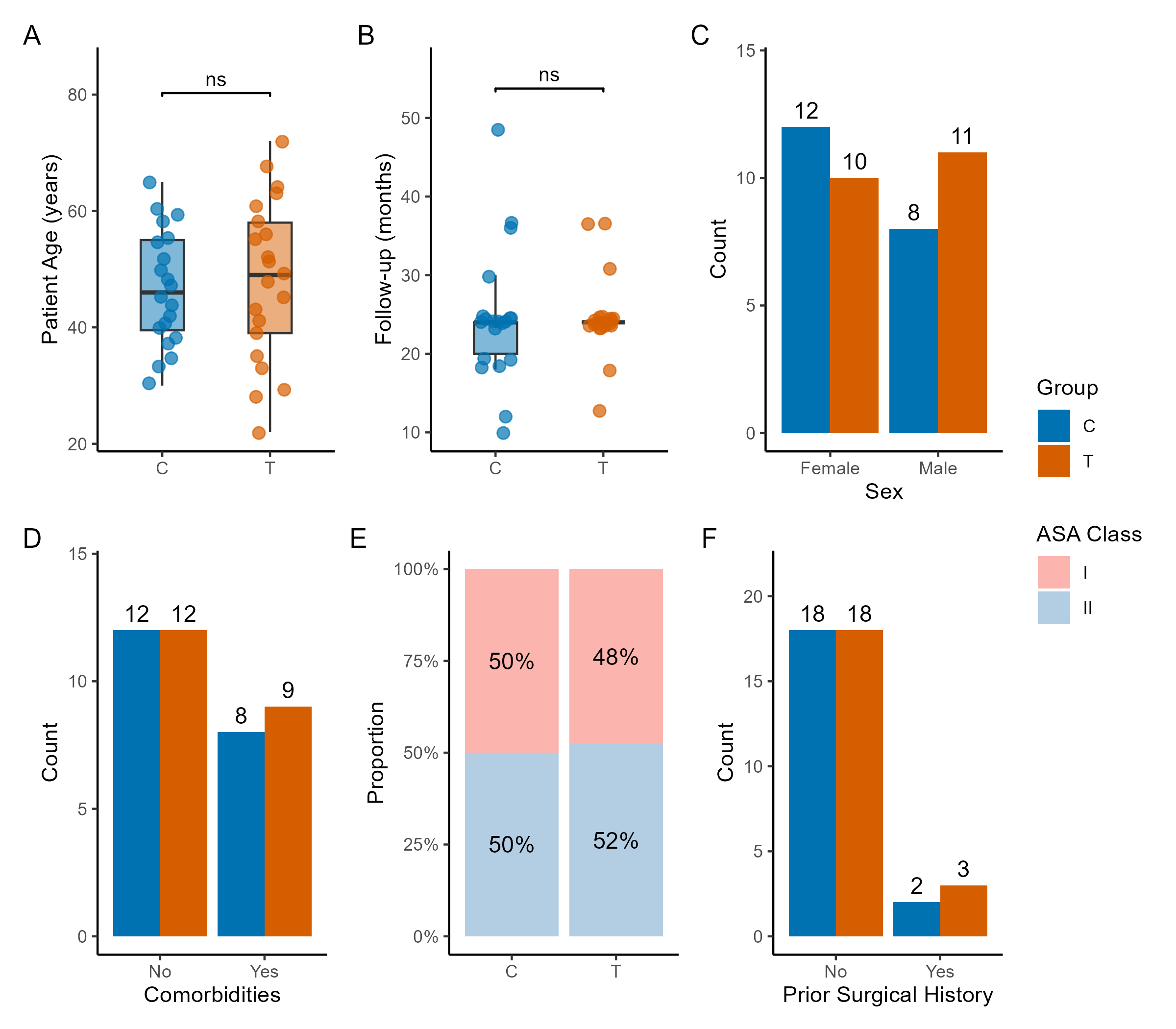

Supplement: Supplementary file 1 — Supplementary material 1. [file 12893_2025_3206_MOESM1_ESM.zip › Fig3.tiff]

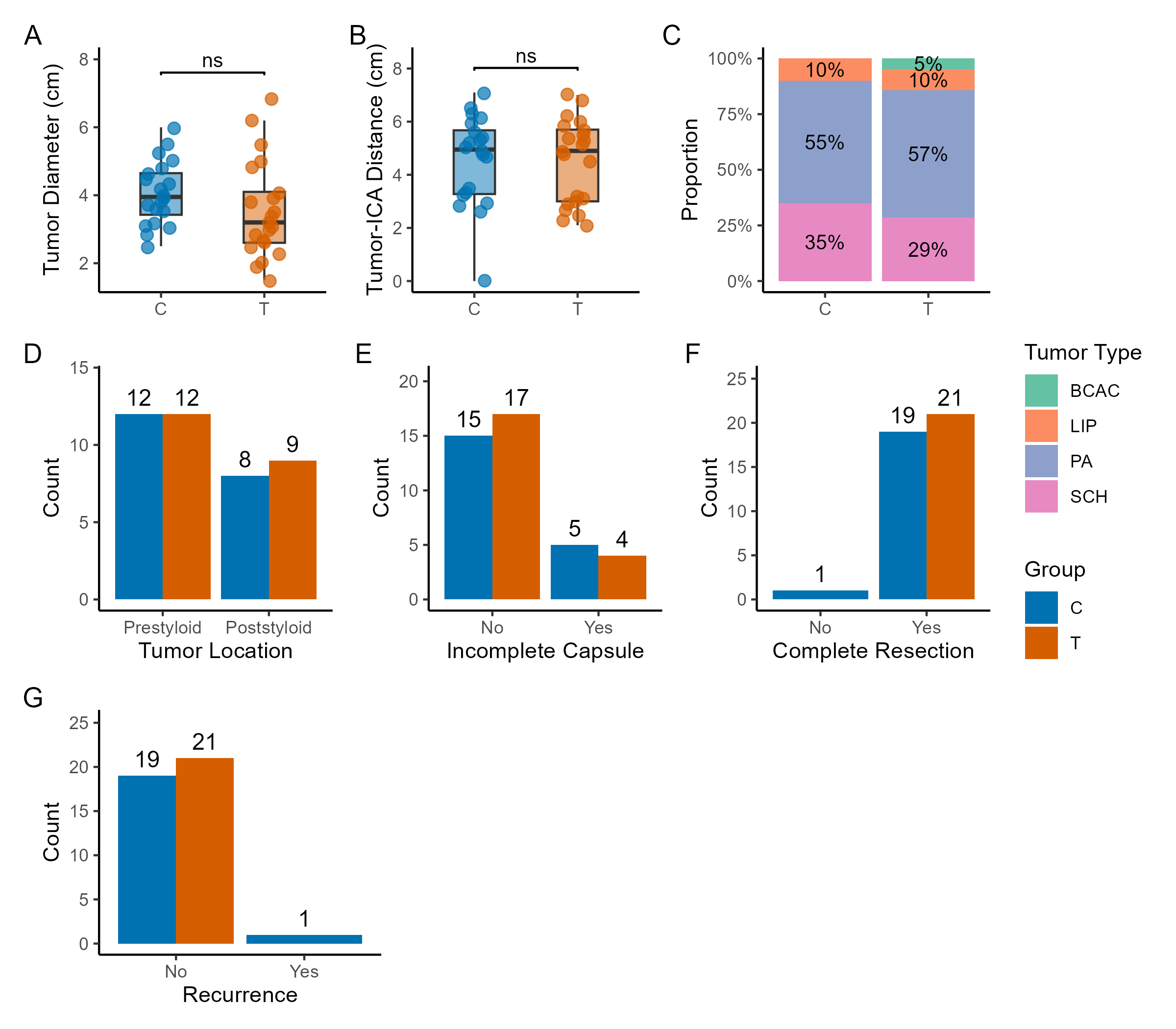

Supplement: Supplementary file 1 — Supplementary material 1. [file 12893_2025_3206_MOESM1_ESM.zip › Fig4.tiff]

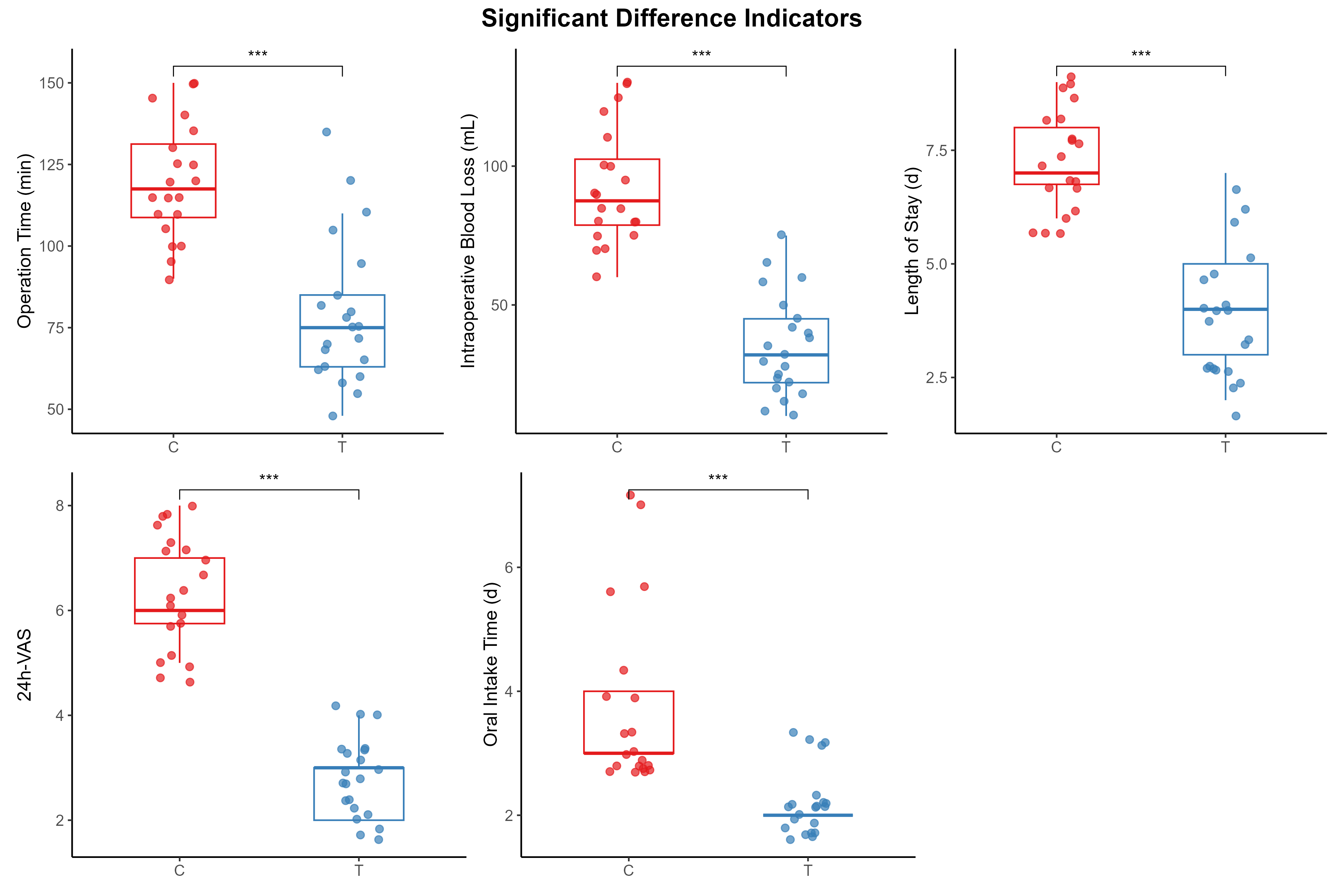

Supplement: Supplementary file 1 — Supplementary material 1. [file 12893_2025_3206_MOESM1_ESM.zip › Fig5.png]

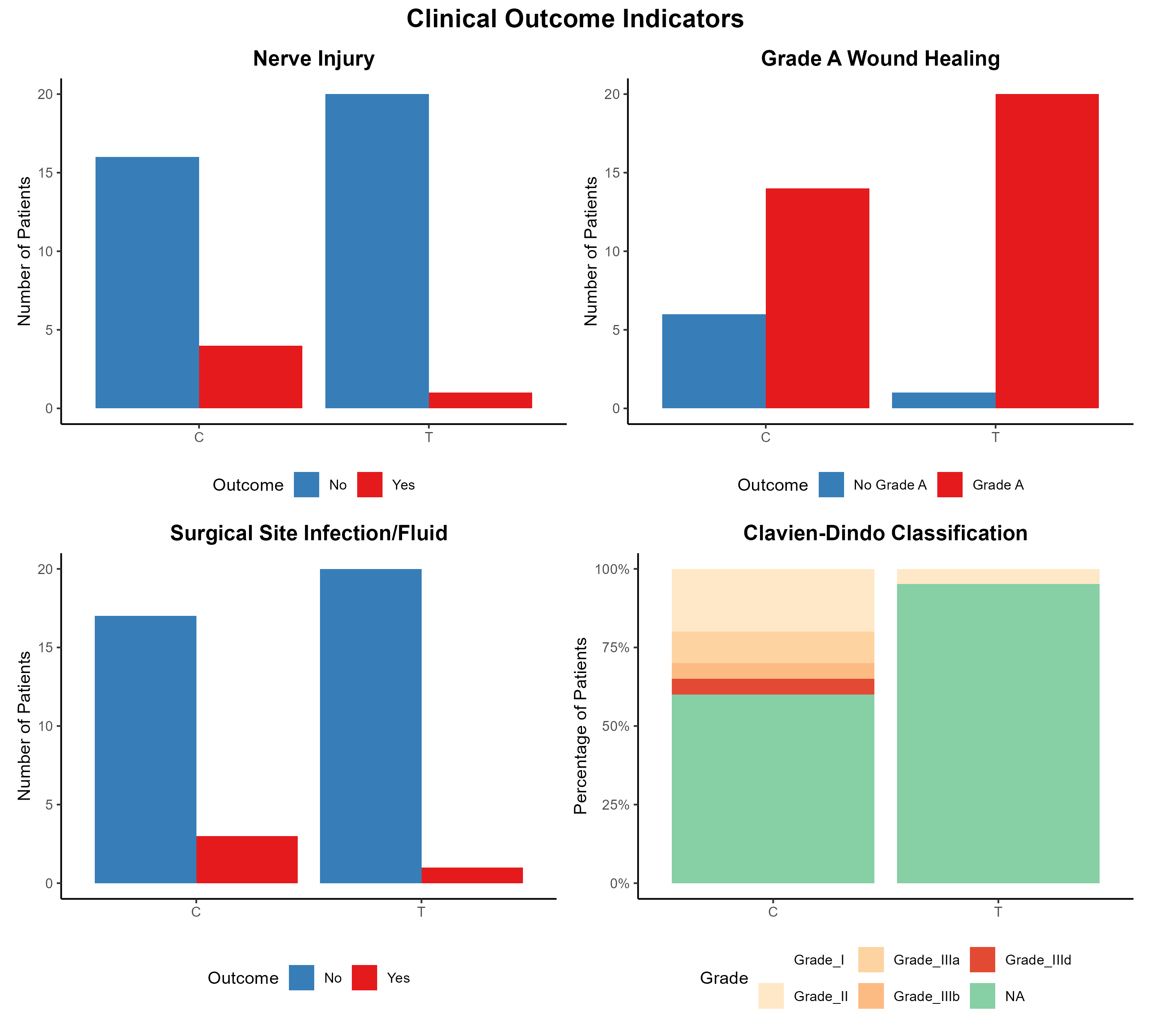

Supplement: Supplementary file 1 — Supplementary material 1. [file 12893_2025_3206_MOESM1_ESM.zip › Fig6.png]

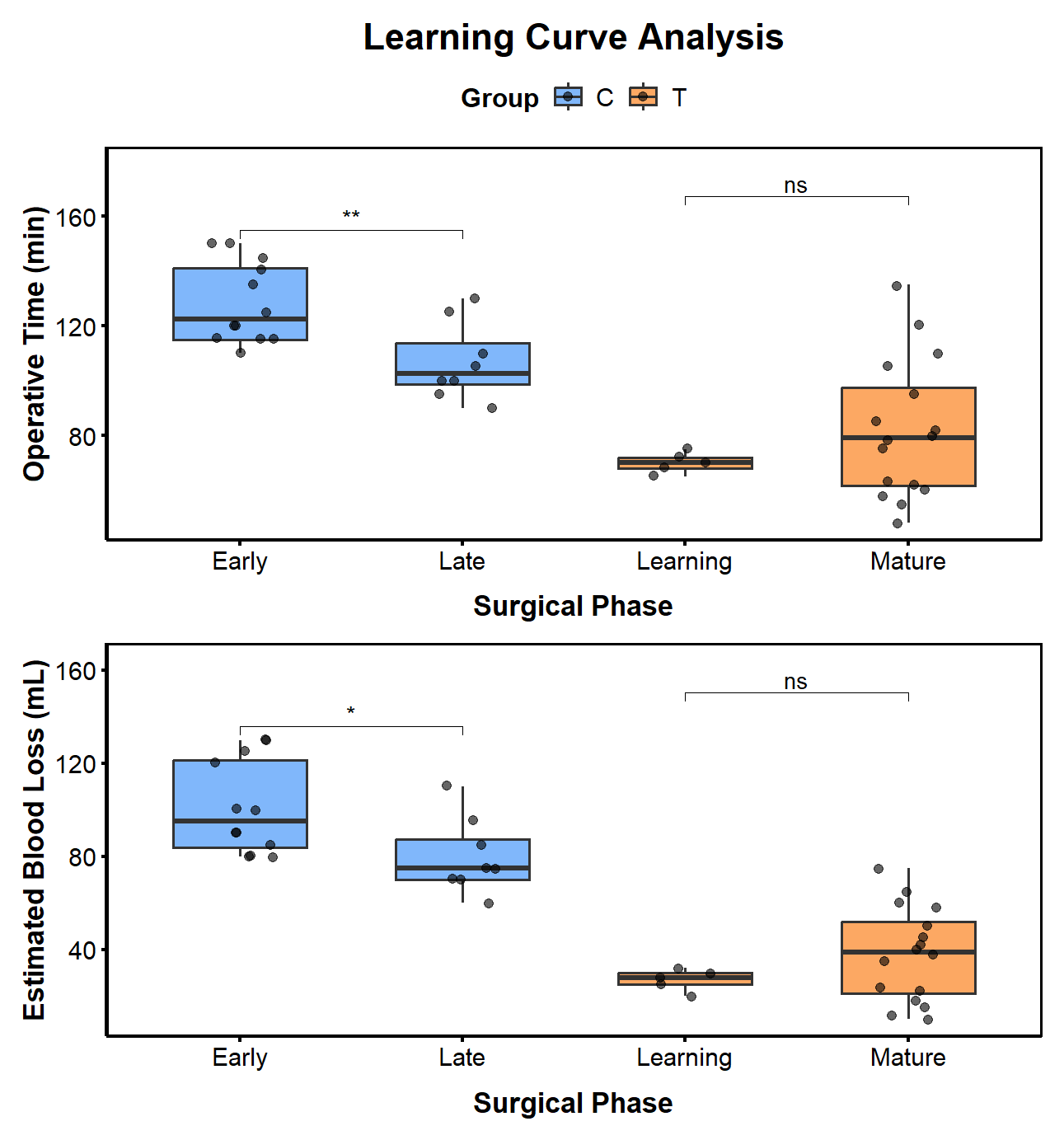

Supplement: Supplementary file 1 — Supplementary material 1. [file 12893_2025_3206_MOESM1_ESM.zip › Fig7.png]

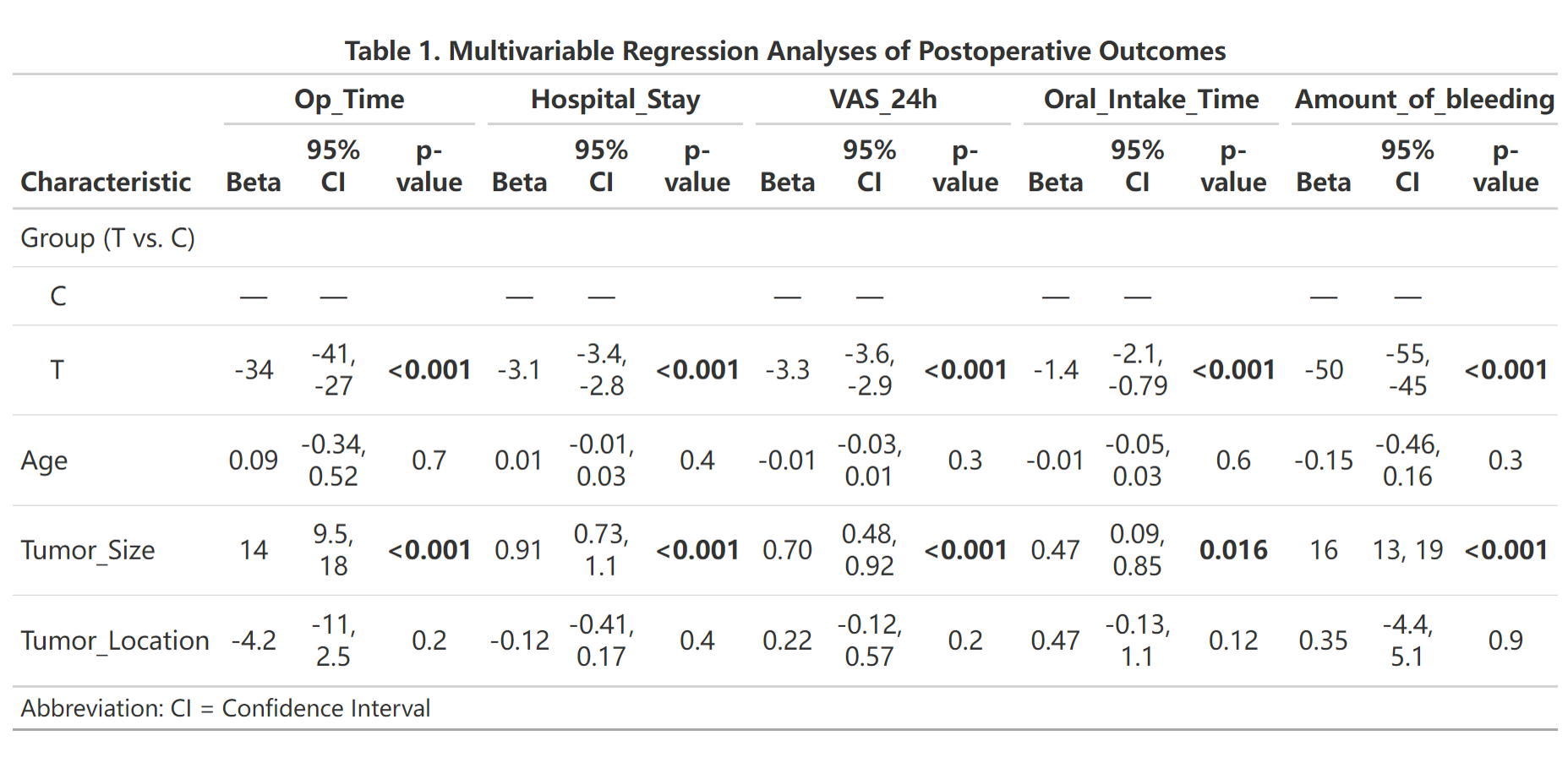

Supplement: Supplementary file 1 — Supplementary material 1. [file 12893_2025_3206_MOESM1_ESM.zip › Fig8.png]

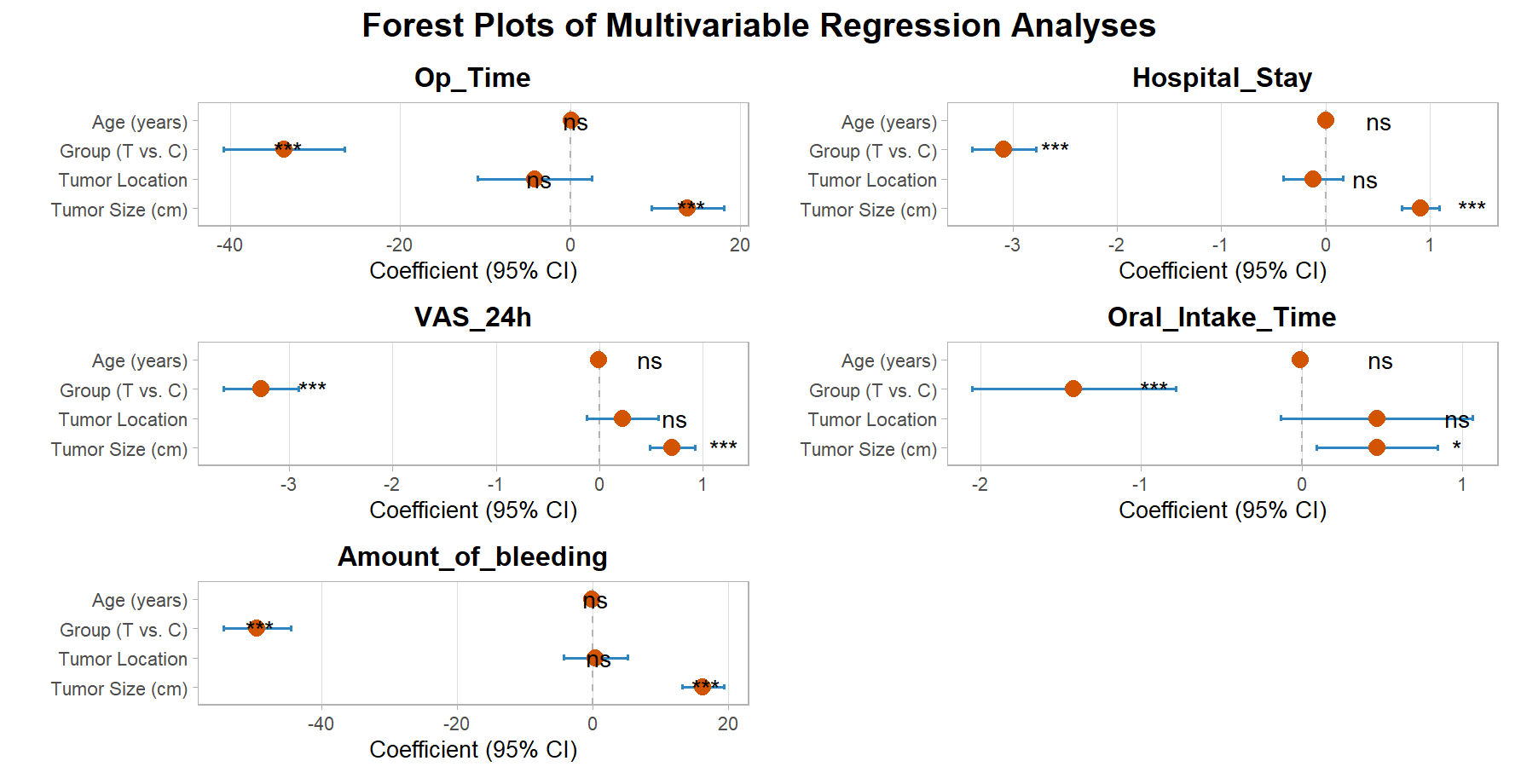

Supplement: Supplementary file 1 — Supplementary material 1. [file 12893_2025_3206_MOESM1_ESM.zip › Fig9.png]

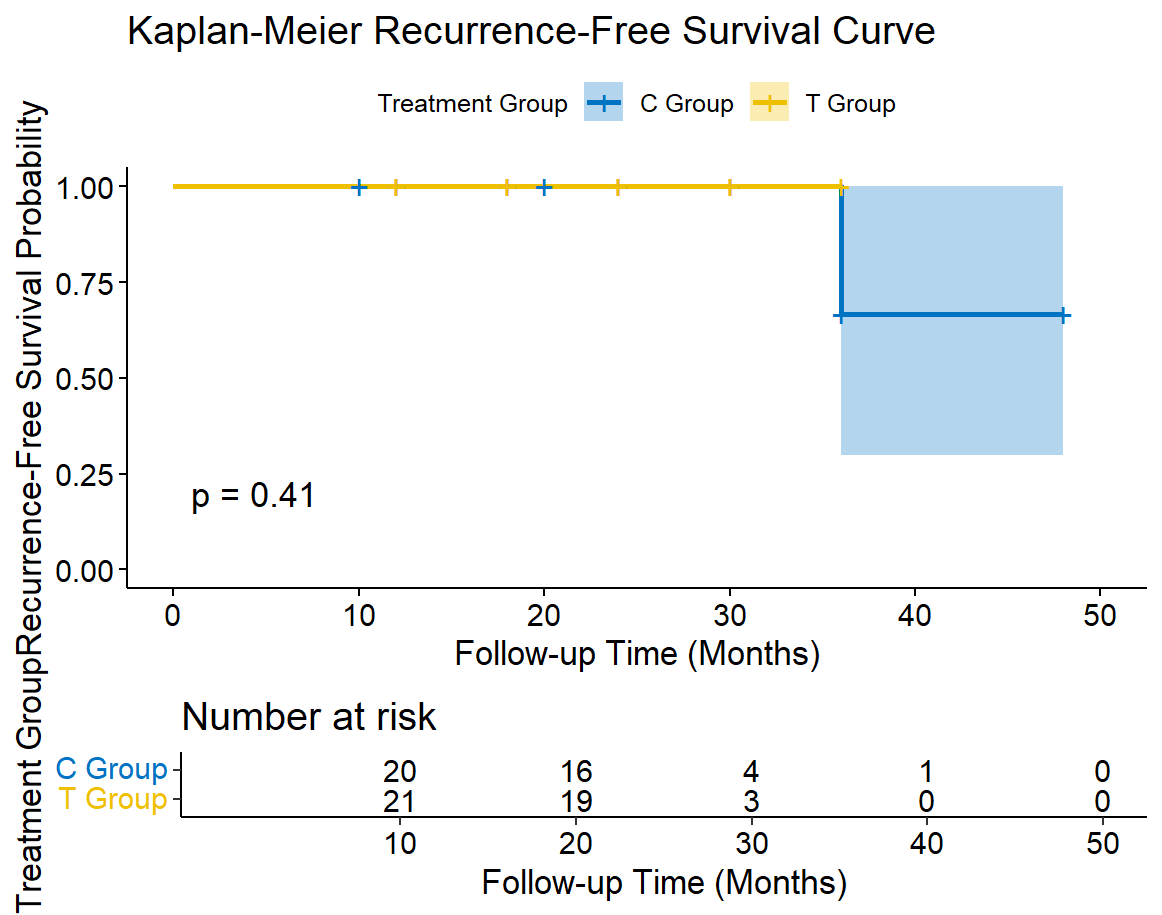

Supplement: Supplementary file 1 — Supplementary material 1. [file 12893_2025_3206_MOESM1_ESM.zip › Fig11.png]

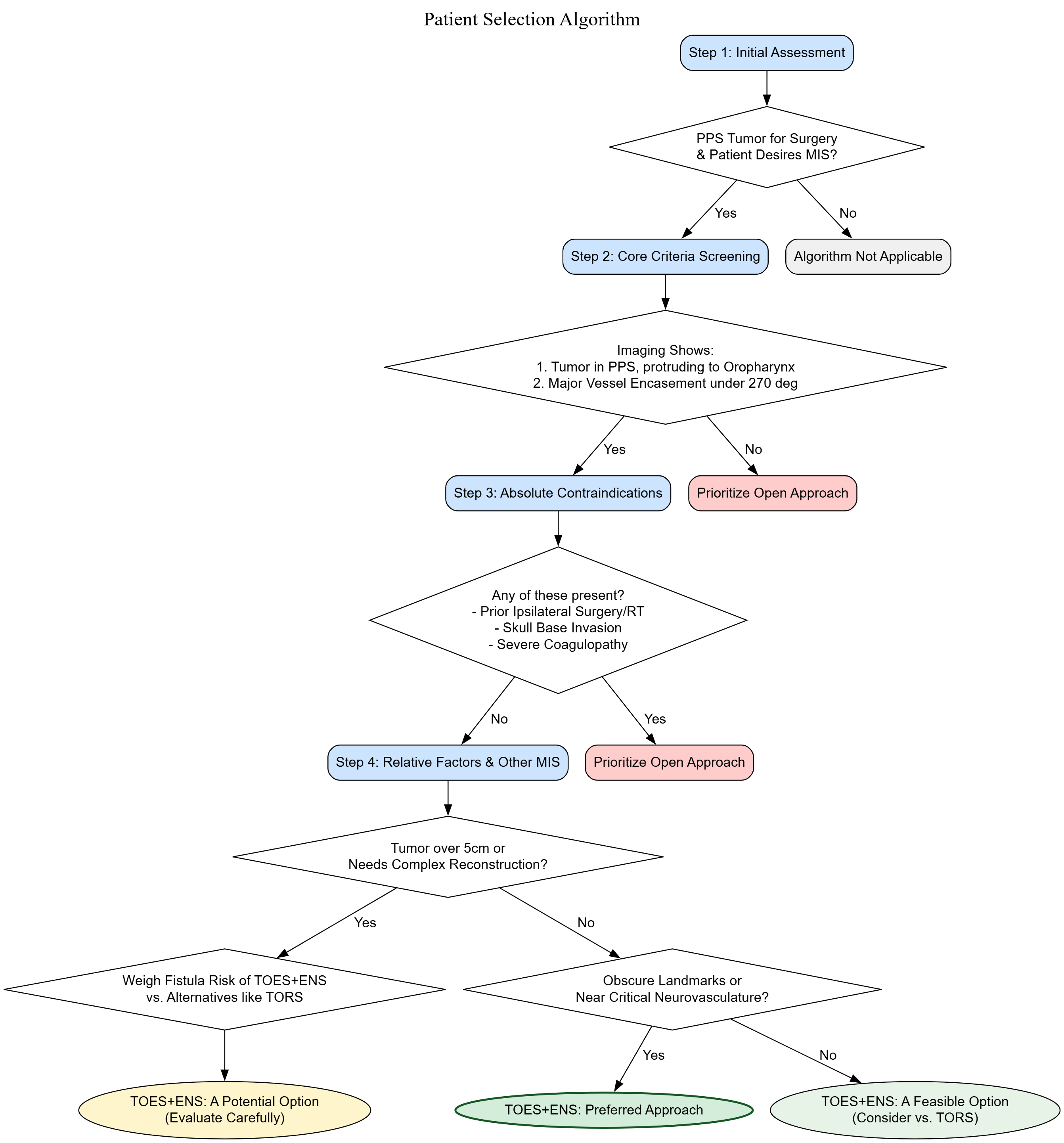

Supplement: Supplementary file 1 — Supplementary material 1. [file 12893_2025_3206_MOESM1_ESM.zip › Fig12.png]

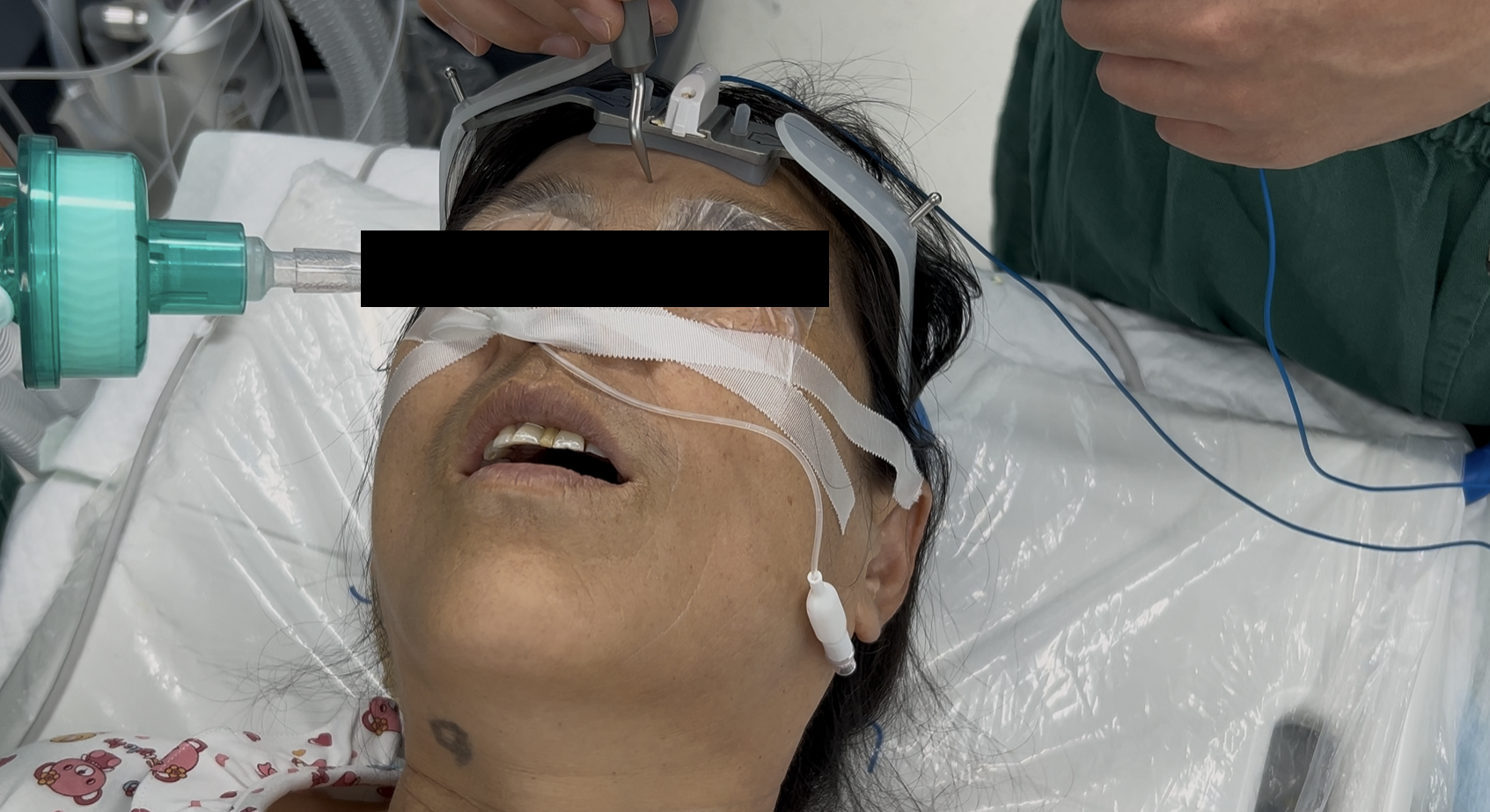

Supplement: Supplementary file 1 — Supplementary material 1. [file 12893_2025_3206_MOESM1_ESM.zip › Supplementary Figure 1.png]

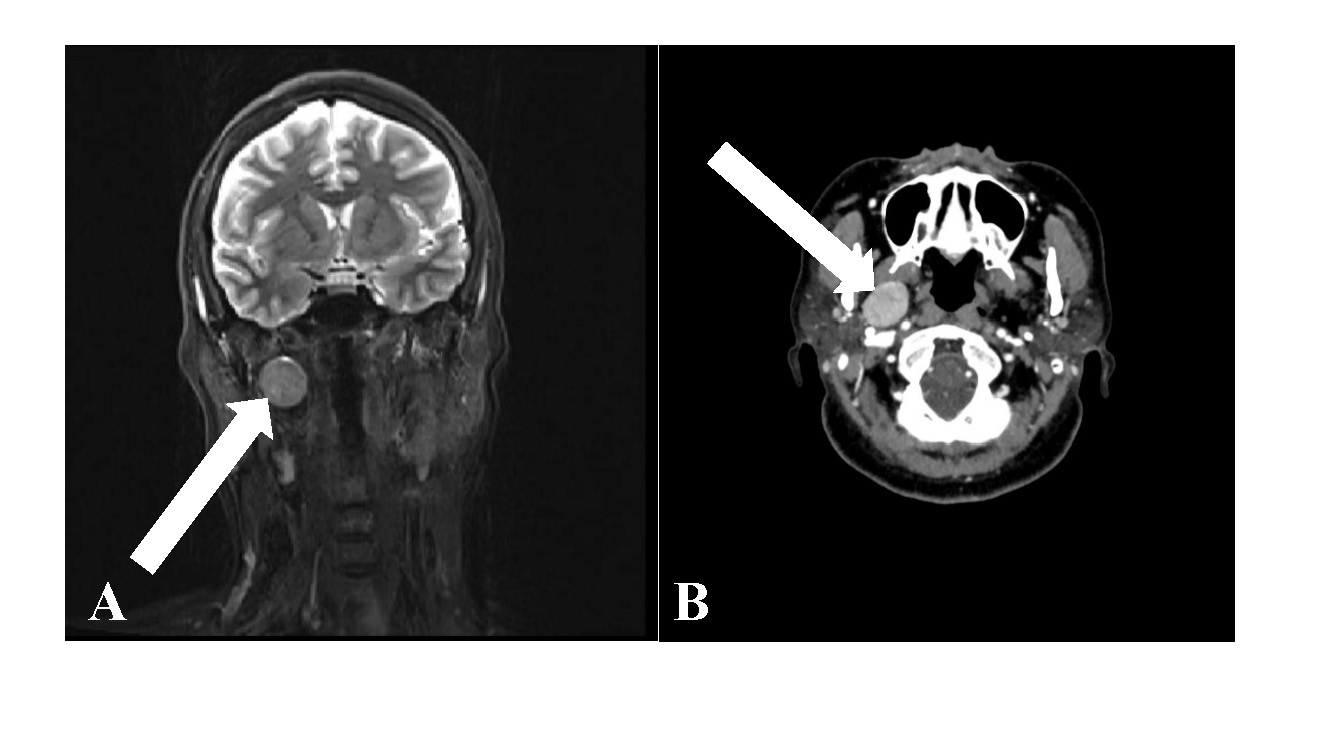

Supplement: Supplementary file 1 — Supplementary material 1. [file 12893_2025_3206_MOESM1_ESM.zip › Fig 1.jpg]

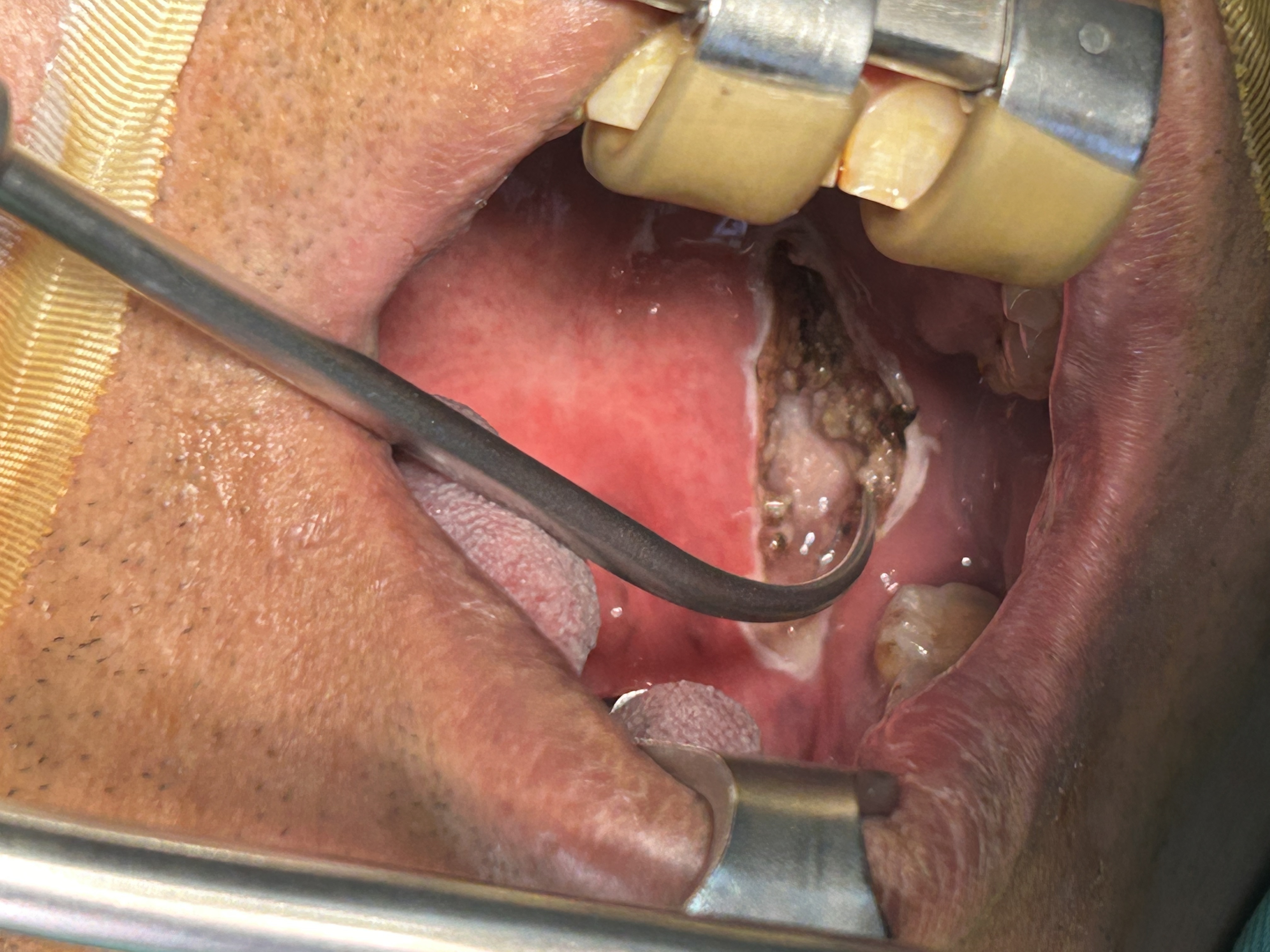

Supplement: Supplementary file 1 — Supplementary material 1. [file 12893_2025_3206_MOESM1_ESM.zip › Fig2-A.jpg]

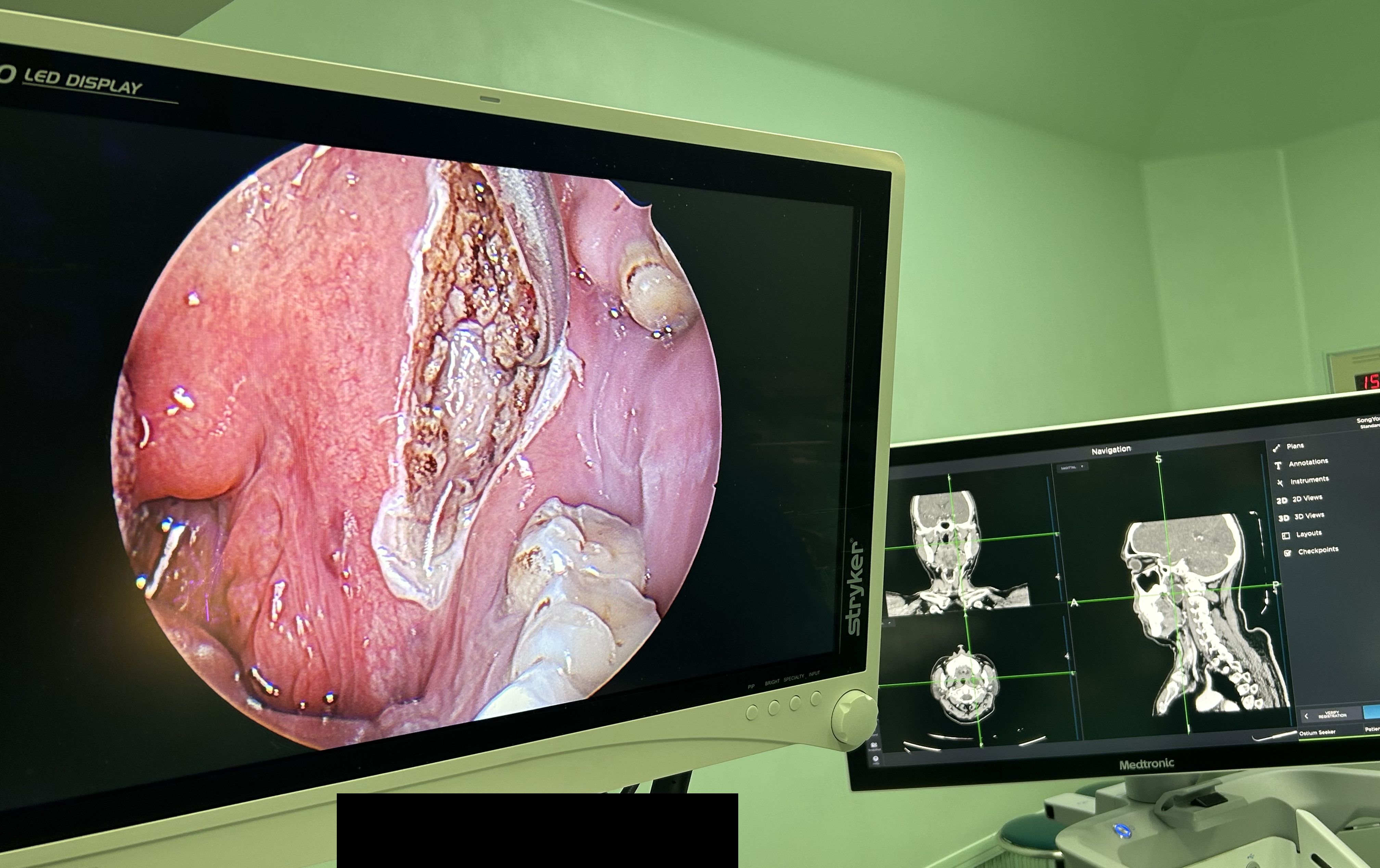

Supplement: Supplementary file 1 — Supplementary material 1. [file 12893_2025_3206_MOESM1_ESM.zip › Fig2-B.jpg]

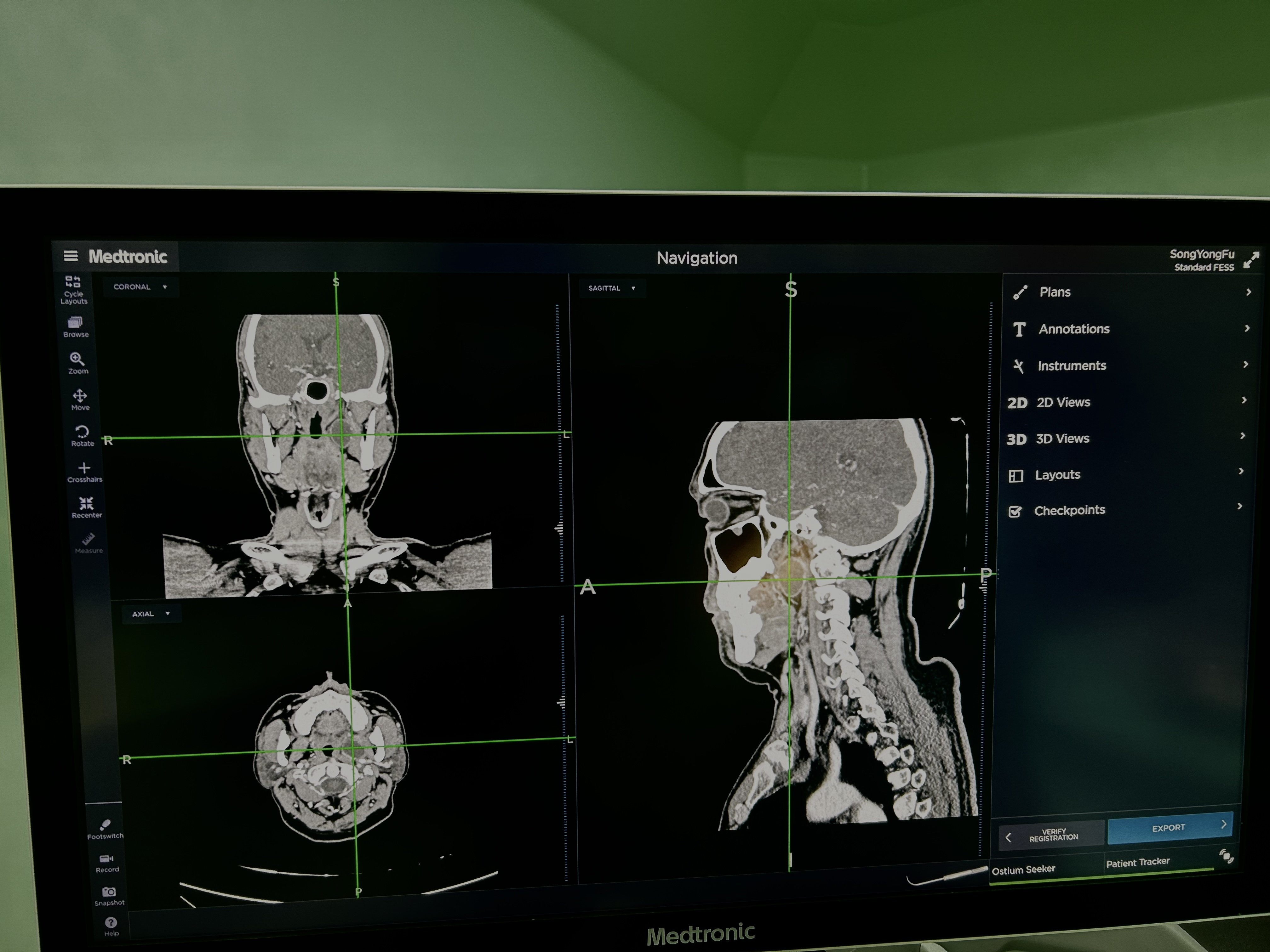

Supplement: Supplementary file 1 — Supplementary material 1. [file 12893_2025_3206_MOESM1_ESM.zip › Fig2-C.jpg]

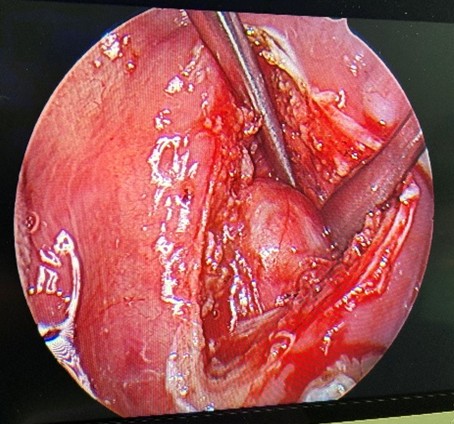

Supplement: Supplementary file 1 — Supplementary material 1. [file 12893_2025_3206_MOESM1_ESM.zip › Fig2-D.jpg]

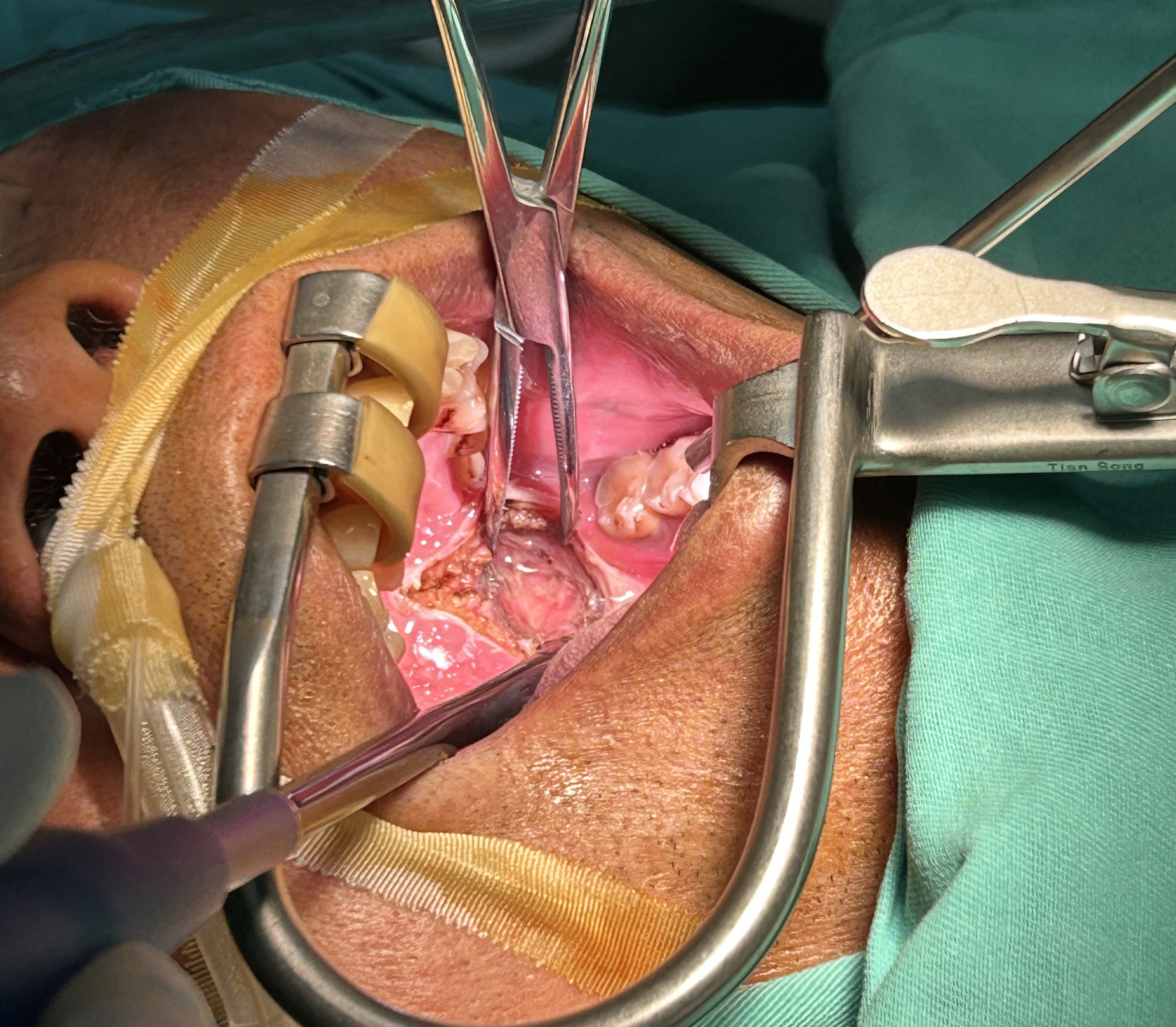

Supplement: Supplementary file 1 — Supplementary material 1. [file 12893_2025_3206_MOESM1_ESM.zip › Fig2-E.png]
